# Place and place names: a unified model

**DOI:** 10.3389/fpsyg.2023.1237422

**Published:** 2023-09-08

**Authors:** Francesco-Alessio Ursini, Yue Sara Zhang

**Affiliations:** ^1^Department of Chinese Language and Literature, Central China Normal University, Wuhan, China; ^2^Department of English, Hainan University, Haikou, Hainan, China

**Keywords:** place, place concepts, place names, place models, discourse representation theory, anchoring relations, formal ontologies

## Abstract

The goal of this paper is to offer a unified account of Place as a central theoretical notion across different disciplines. We show that while psychology, geography and other sciences have been converging to a unified view of this notion, linguistics still offers a fragmented perspective. Consequently, place names lack a full-fledged analysis that connects this category to the psychological concept of place. We propose to overcome this *impasse* by introducing a multi-modal Discourse Representation Theory (DRT) account of place as a conceptual construct and place concepts as specific instances of this construct. We show that current variants of DRT permit us to model place names and their senses, i.e., the meaning(s) that individuals associate with *Sydney*. We then model non-linguistic place concepts, i.e., the mental representation(s) that individuals can have of the city carrying this name. We present a model of the relation between linguistic meaning and conceptual content via the notion of anchoring relations applied to place. We pair this formal treatment with a morpho-syntactic account of place names building on current generative syntax treatments of proper names. Once we have a morpho-syntactic and semantic model of place names, we use a frame semantics treatment to account for lexical relations among place names. We test the overarching model on a set of recalcitrant problems afflicting current linguistic and multi-disciplinary treatments of place. These are the grammatical complexity and lexical content of place names, place concepts and their networks, and inter-subjective, communicative models of place in discourse. By solving these problems, our account integrates several frameworks (DRT, conceptual analysis, generative syntax, frame semantics) and connects several disciplines (linguistics, psychology, geographic information science, communication models) via a novel, multi-modal account of place. We conclude by discussing the theoretical and empirical import of these results.

## 1. Introduction

*Place* is often defined as any location that can welcome humans and their social interactions, and to which humans can develop forms of attachment (Cresswell, 2014: Ch. 1). Contemporary geographical thinking on place originated in Tuan (1977) and deeply influenced psychology (Manzo and Devine-Wright, 2021), sociology (Low, 2016), and other disciplines. Complex theories of place(s) and relations between individuals and these concepts therefore emerged (e.g., Scannell and Gifford, 2014). Formal treatments of the properties shaping places were proposed in psychological frameworks (Canter, 1977, 1997). Formalizations of these notions also emerged in Geographic Information Science (GIS) and exploited *formal ontologies*, first order logic-based specifications of conceptual domains (Hamzei et al., 2020). Place is thus an increasingly refined notion that finds a mentalist counterpart in the general concept of “place,” and in its more specific declinations (e.g., “country,” “city”).

Linguistic disciplines, however, present a different picture. Toponomastics studies place names and their etymological roots (e.g., *Sydney, Pitt Street*: Tent and Blair, 2011; Perono Cacciafoco and Cavallaro, 2023). Linguistic typology offers cross-linguistic analyses of place names’ grammatical properties (Stolz and Levkovych, 2020). Though both frameworks have made important discoveries on place names, neither framework connects these discoveries to theories of place. Furthermore, the conceptual semantics and generative syntax frameworks use the term “Place” to analyse adpositions and their senses (respectively Jackendoff, 1983; Svenonius, 2010). The consequences of this theoretical mismatch between linguistics and other disciplines create the empirical *impasse* that we illustrate via (1)–(3).

(1) *The plane landed in Sydney*.
(2) *Pitt Street is in Sydney.*
(3) *The boys are playing in a park*.

The sentence in (1) describes an event of a plane landing in the Australian city of Sydney; (2) describes a certain street, as a place within Sydney’s business district. *Sydney* and *Pitt Street* are the place names that allow individuals to refer to these places. Instead, (3) describes some boys playing in a nameless park, i.e., a location introduced in discourse without a specific “platial” (i.e., place-like, Tenbrink, 2020) status. Therefore, (1)–(2) are sentences offering semantic information about two closely related and unique places; (3) offers information about some non-specific location identified as a park.

Geographic and psychological frameworks suggest that (1)–(2) convey information about places via place names, mediated via mind-internal place concepts (Kuhn, 2012; Low, 2016). Instead, most toponomastic works suggest that this reference is direct: individuals do not have concepts about places (Coates, 2017). Generative and conceptual frameworks would consider the P(repositional)P(hrase)s *in Sydney* and *in a park*, in (1)–(3), as equally referring to places via place concepts (Svenonius, 2010). Linguistic frameworks do not attempt to capture the content that place names carry; some frameworks do not even semantically distinguish place names from other NPs and spatial PPs. Hence, place names and place concepts receive short thrift in linguistics.

This neglected status of place names in linguistics has inter-disciplinary ramifications. Psychological research suggests that individuals can develop rich place concepts, and associate them to place names (Scannell and Gifford, 2010; Davies, 2013). Some individuals may use (1)–(2), and thus the name *Sydney*, to describe situations and facts about a city they love; others, for a place they are unfamiliar with. Other uses are possible; individuals can express their own conceptual views about places via place names, and continually develop rich mind-internal representations (i.e., concepts) of places. GIS works instead suggest that place names retrieval algorithms need detailed semantic and morpho-syntactic representations to inform place models (Buscaldi, 2011; Zhang and Gelernter, 2014). Linguistic theorising on place names, as we gleaned via (1)–(3), lacks a morpho-syntactic analysis of place names, and mostly explains away their semantic properties. Therefore, the cognitive mechanisms by which place names convey information about places remain mostly unexplored.

The goal of this paper is to ameliorate this situation by proposing a linguistic model of place names integrated with a conceptual model of place. We use *Discourse Representation Theory* (DRT: Kamp et al., 2011) as a semantic framework to model the content of *Sydney*, *Pitt Street*, and other place names. We choose this framework because current DRT architectures use a general format (“Discourse Representation Structures”) to model *any* mental content, irrespective of the modality (e.g., Pross, 2010; Maier, 2015; Kamp, 2022). Therefore, DRT provides a formal language that can model the multi-modal cognitive content of place and place names into a general, if preliminary theory of “place.” We integrate DRT as a *Domain*-general Representation Theory with a generative analysis of place names. We then show that the lexical properties and relations connecting place names can be modelled by integrating this multi-modal model (DRT plus syntax) with a frame semantics analysis. We therefore show that our model of “place” can pave the way for a novel, inter-disciplinary approach to platial research (Tenbrink, 2020).

To reach this goal, we first analyse “place” as a psychological concept of a geographical notion (Section 2). We then discuss GIS formal treatments (Section 3). We problematize the uses of the label “place” in Linguistics and outline *desiderata* for a linguistic analysis (Section 4). We develop a multi-modal, representational model of place concepts and place names. We achieve this result via domain-general DRT’s anaphoric relations between cognitive domains (“anchoring relations”), coupled with a generative view of place names’ grammatical properties. We then address a number of recalcitrant problems stemming from the lack of a theory of place names informed by theories of place. We thus offer an account of the linguistic properties of place names, how they represent place concepts and networks, and how individuals can share place concepts and relations in discourse and communication (Section 5). Section 6 discusses the consequences of this proposal for a theory of place. Section 7 concludes.

## 2. Place as a concept: from geography to psychology

The goal of this section is to discuss how psychology has absorbed the geographical notion of place. First, however, we establish our terminology. We use the term *place concepts* to refer to concepts for cities, streets and other entities that can be considered particular instantiations of the general concept of *place*. “Sydney,” “Pitt Street,” are possible labels for *singular* place concepts: concepts for one (quantity-wise) unique place. We standardly conceive concepts as multi-modal, mind-internal representations about entities in the world (Laurence and Margolis, 1999; Margolis and Laurence, 2007; Carey, 2009; Ursini and Samo, 2022). We use *place(s)* as a label for geographic entities, the mind-external counterparts of Place concepts. We use italics for place names: *Sydney*, *Pitt Street, respectively*, are the names for the “Sydney,” “Pitt Street” place concepts. Given this distinction between mind-internal place concepts and mind-external places, we will discuss how reference to either entity type can come about in Sections 4–5, once we introduce the relevant notions.

The emergence of place as a theoretical construct originates in the work of geographers offering philosophical reflections on humans and their environment (Relph, 1976; Seamon, 1979; Tuan, 1979, 1990). These geographers suggested that humans develop place concepts via their interaction with places, thus imbuing these concepts with subjective meaning. Subsequent works sought to explore the psychological and social dimensions that could shape these concepts (Couclelis, 1992; Agnew, 1993; Harve, 1994; Thrift, 1999; Hollenstein and Purves, 2010). Hence, they suggested that the content of place concepts can develop and vary considerably over time, depending on individuals and groups’ emotive and cognitive connections to places. For instance, local dwellers can develop love for Sydney as their home; international rugby fans can love or hate this city when visiting it to experience rugby matches; manyfold possible forms of attachment are certainly possible. Current theories in geography (e.g., Agnew, 2011), philosophy (e.g., Malpas, 2018) and psychology (e.g., Smith, 2017) thus accept place and place concepts as mirror-like notions about locations that are meaningful to humans.

Research on place concepts abounds (cf. Agnew et al., 2015: Ch. 1; Mocnik, 2022); however, proposals tend to diverge on working terms and notions. The notion of “place attachment” has received the most attention in psychology (Smith, 2017; Manzo and Devine-Wright, 2021), sociology (Low and Altman, 1992; Low, 2016) and anthropology (Fitch, 2014; Harun et al., 2015), among others. Place attachment studies investigate how groups and individuals can develop forms of attachment to places by dynamically fostering (place) concepts. Attachment relations can involve manifold emotional, social and cognitive factors interacting in complex manners (e.g., Lewicka, 2008, 2011). These relations can be shaped via direct experience of a place or via indirect information gained via multi-modal sources (e.g., respectively Tang et al., 2021; Merciu et al., 2022). For instance, Sydney locals and tourists can develop attachment via experiences, alone and in groups (e.g., as rugby fans), and via books, videos, social media and any other relevant media.

Though place attachment studies may abound, a dearth of formal models seems attested (cf. Turton Catherine, 2016; Inalhan et al., 2021). One exception is the “tripartite place attachment” model of Scannell and Gifford (2010, 2014, 2017). In this model, place attachment is a triadic relation between “People,” “Places” and “Processes.” In the “People” domain, the model studies whether individuals or groups develop forms of place attachment (cf. also Manzo, 2005). In the “Places” domain, the model studies the *facets*, i.e., features and dimensions of variation constituting place concepts (Canter, 1977, 1997, 2012). Place facets can represent social and physical properties of places as two distinct types (Uzzell et al., 2002). In the “Processes” domain, the model studies attachment relations between individuals and places via emotive, cognitive and social bonds (Brown et al., 2003). The model suggests that place attachment arises when people (e.g., local inhabitants) develop relations towards places (e.g., love for Sydney) via processes (e.g., living “there”), and via place concepts.

Other notions attaining to place have also been thoroughly investigated, such as place identity, place memory, place formation, and others falling under the “place-making,” “sense of place” rubrics (Seamon, 2018). Place identity studies, for instance, investigate how individuals can shape their identities via the places they interact with (Proshansky et al., 1983; Hay, 1998; Pretty et al., 2003; Banini and Ilovan, 2021). These studies also investigate how the physical and social features of places shape the identities of individuals and the groups they belong to (Bonaiuto et al., 1996; Twigger-Ross and Uzzell, 1996; Stedman, 2002; Clayton, 2003; Knez, 2005). For instance, Sydneysiders can define themselves as city dwellers and beach-goers because Sydney is an Australian city blessed with several beaches. In general, individuals internalise places’ features as mental facets shaping place concepts that they use to develop complex place attachment relations. Place-making is therefore an ever-going process by which individuals imbue place concepts with meaning, thus defining their “sense of place” via the facets constituting these concepts (Seamon, 2012, 2018, 2021).

As our necessarily concise summary suggests, psychological theories of place take a dual stance inherited from Geography (Cresswell, 2014). While “place” describes mind-external entities, place concepts describe internal, multi-faceted and subjective representations that individuals have of places (e.g., Marzano and Siguencia, 2016). Psychology and other cognitive disciplines usually propose model-specific terms and assumptions about place concepts (Seamon, 2018). However, they converge in assuming that place concepts reflect social and physical/geographical aspects of places via facets, and guide the formation of place attachment relations (Scannell and Gifford, 2014). Furthermore, facet theory analyses suggest that place facets can be operationalised via formal languages such as first order logic (e.g., Canter, 1977, 1997, 2012). However, they leave the development of these tools mostly underdeveloped. Psychological models thus offer formally vague models of place concepts. At the same time, they also generally gloss over linguistic matters.

## 3. GIS and formal ontologies of place concepts

The goal of this section is to analyse how GIS has developed formally explicit treatments of place concepts. Within this field, the development of place models has moved in parallel with the exponential growth of information in two domains. One includes on-line gazetteers and geographical databases (Abdelmoty et al., 2007; Baglioni et al., 2007; Jones et al., 2008; Winter et al., 2009; Allen et al., 2016). The other includes “volunteered geographic information” (Goodchild, 2007; Keßler et al., 2009; Southall et al., 2011; Sui and Goodchild, 2011; Derungs and Purves, 2014; Dunn, 2017). On-line, open-access gazetteers and social media (e.g., OpenStreetMap, FourSquare) exist thanks to the spontaneous (“volunteered”) information that users offer about Places (Williams et al., 2012; Janowicz et al., 2013; Gao et al., 2017; Hu et al., 2019). GIS studies build models to represent these views as expressions of individuals’ place concepts and their inter-subjective variation.

Researchers have hence developed *geo-ontologies*, structured databases in which platial, individual-based content is explicitly represented. Most works use variants of first order logic (Frank, 2003; Agarwal, 2005a,b; Bishr and Kuhn, 2007; Fonseca, 2008; Fonseca and Câmara, 2009; Couclelis, 2010; Vasardani and Winter, 2016). Furthermore, several proposals use “geo-atoms” as the building blocks of these ontologies (Kuhn, 2005, 2012; Goodchild et al., 2007; Winter and Freksa, 2012; Hu, 2018; Purves et al., 2019). The core properties of geo-atoms can be defined as follows. First, geo-atoms are based on objects, usually defined via crisp boundaries. Second, geo-atoms represent the locations of these objects defined via a spatio-temporal coordinate system. Third, geo-atoms represent the properties and corresponding values associated to objects and locations. The use of geo-atoms allows researchers to represent place concepts via the format in (4)–(5) (cf. Kuhn, 2012).

(4) *A:<x,l,S(l(x))>*
(5) *A:<Sydney,l,S = {extension,number-of-districts, popularity, citizens’-opinion},S(l(Sydney))>*

As (4) shows, a geo-atom *A* represents an object *x*, a spatio-temporal location *l* for the object, a set of properties *S* individuating the object, and their respective values for the object at its unique location [i.e., *S(l(x))*]. The location function *l(x)* represents a “geo-object”: an object occupying a single spatio-temporal location. Consider now (5): this geo-atom offers a compact formalisation of a possible singular place concept for Sydney. For instance, Sydney can have 5 million inhabitants and a certain number of districts. It can also be popular among tourists and be a great or awful city to live in, according to its citizens. These and other properties can represent how individuals may develop place concepts for places, and connect these concepts into complex conceptual networks (Hu, 2018; Purves et al., 2019).

Research on GIS ontologies has also focused on two sub-goals: the formalisation of place and place concepts’ properties, and of relations among places (Ballatore et al., 2014; Ballatore, 2016). For instance, Hamzei et al. (2020) formalises facets as the properties and corresponding values that allow individuals to individuate places in space and time (cf. also Entrikin, 1994, 1996, 1997; Canter, 2012). This work divides place concept facets into two macro-types: geographic and anthropocentric, i.e., affordance-centred, facets (Gibson, 1979; Egenhofer and Mark, 1995; Galton, 2010; Jonietz and Timpf, 2015). Both facet types find their justification in volunteered descriptions of Places. For instance, WeChat users can tag pictures of Sydney’s Pitt Street and describe it as a peaceful central district street, providing information on geographical (i.e., location) and anthropocentric (i.e., peacefulness) facets they associate to this street. Representational systems such as geo-atoms can therefore represent place concepts, and can also represent facets as formal properties shaping these atoms [e.g., *extension* in (5)].

GIS works have also introduced the notion of “place network,” which requires some motivation for its current discussion. Several works have suggested that the formal modelling of concepts also passes through the study of relations (spatial, conceptual) among Places (Montello, 1993, 2009; Golledge, 2002; Montello et al., 2003; Tenbrink and Kuhn, 2011). Recent works on place information retrieval have shown that geo-tagged sources often offer information about “what” facets individuate places, but also “where” these places can be located (Vasardani et al., 2013; Zhou et al., 2016; Chen et al., 2018; Hamzei et al., 2021). For instance, Instagram users can tag pictures of Pitt Street and Hyde Park while being in Sydney. They therefore provide information about this street and this park being “in” Sydney, and about Sydney being a place that includes other distinct places. The place concepts and models that individuals can share in discourses must represent these manifold relations (Chen et al., 2018; Maren et al., 2020).

These insights are summarised in Purves et al. (2019)’s “ontological commitment” about place concepts. Formalisations of place concepts (e.g., geo-atoms) must represent objects occupying locations and instantiating manifold facets (i.e., places); they can enter relations and participate in events (i.e., define networks). To see why this ontological commitment finds empirical justification, consider (6)–(7) and how they convey information about two places, Sydney and Brisbane.

(6) *Sydney is South of Brisbane*.
(7) *Sydney and Brisbane always had a cultural and sportive rivalry.*

The sentence in (6) suggests that Sydney and Brisbane stand in a (approximate) “South-of” spatial relation. Instead, (7) suggests that the two Australian cities are connected via a type of social relation: the “rival-of” relation. Sports teams and musical bands have participated in various events that can have motivated the growth of this rivalry over time. Individuals who offer such descriptions of these places thus also offer information about their “Sydney” and “Brisbane” concepts, and how they subjectively connect these concepts. Hence, Purves et al. (2019)’s commitment can be interpreted in this way. Place concept formalisations (e.g., geo-atoms for “Sydney,” “Brisbane”) must represent objects (e.g., the physical cities) and their properties in their locations. These concepts can enter relations (e.g., being “South of” another place), and participate in events (e.g., “being in a rivalry”). Crucially, (6)–(7) also suggest that place names and other categories (e.g., adposition *South of*) introduce reference to these concepts. However, place names refer to places; spatial adpositions, instead, refer to relations between places and/or locations.

## 4. “Place” in linguistics

The goal of this section is to assess the status of “place” notions in linguistics; we begin our analysis from Toponomastics. Most works focus on the etymology of place names, and practices of use among communities (Kadmon, 2000; Watts, 2004; Ainiala et al., 2012: Ch. 2). Studies investigating place names’ grammatical properties usually suggest that place names can include *generic terms* and *specific terms*. While *Street* is a generic term, *Pitt* in *Pitt Street* is a specific term (Zinkin, 1969; David, 2011; Tent and Blair, 2011; Blair and Tent, 2015, 2021; Tent, 2015; Perono Cacciafoco and Cavallaro, 2023). Some works argue that place names represent individuals’ conceptualisation of their environment (Reszegi, 2012, 2020a,b). Most works however propose that place names only introduce “proper” (i.e., pragmatic) reference conditions to mind-external places (Coates, 2006a,b, 2013, 2017; after Kripke, 1972). Hence, toponomastics studies mostly ignore place concepts and their relations to place names.

Typology has instead uncovered three key morpho-syntactic properties of place names across languages (Cablitz, 2006; Nash, 2015; Nühling et al., 2015; Rybka, 2015). First, place names only including specific terms act as bare NPs (e.g., *Sydney*). Place names including generic terms are phrasal compounds (e.g., *Pitt Street*; Schlücker and Ackermann, 2017; Schlücker, 2018, 2020). Second, place names usually refer to unique discourse entities, and thus incorporate definiteness and specificity features (Anderson, 2004, 2007). Hence, they are a highly distinctive NP sub-type across languages (Van Langendonck, 2007, 2017; Köhnlein, 2015; Van Langendonck and Van de Velde, 2016). Third, place names often combine with case markers (e.g., locative case in Finnish) and may license the omission of spatial adpositions (Stolz et al., 2017; Stolz and Warnke, 2018; Haspelmath, 2019; Stolz and Levkovych, 2020). Typological studies thus offer fine-grained grammatical analyses of place names but explain away their semantic properties, and their relation to place concepts as psychological constructs.

While toponomastics and typology are frameworks that focus on functional, social and thus mind-external uses of languages, conceptual semantics and generative syntax take a mind-internal stance. Conceptual semantics attempts to individuate the core conceptual categories underpinning the senses of linguistic units (Jackendoff, 1983, 1990, 1997, 2002, 2010; van der Zee, 2000; van der Zee and Slack, 2003). The framework suggests that adpositions (e.g., *in*, *behind*) find their senses in PLACE-functions, which map the senses of NPs, i.e., concepts for objects, to concepts for locations: “Places,” in this framework. For instance, the PP *in a park*, from (3), includes an adposition, *in*, and an NP, *a park*. The sense of the NP is a concept/function individuating a park in discourse, as a landmark object. The sense of *in* is a concept/function mapping the “park” concept to an internal location, i.e., an “in” place. Other functions (e.g., “front” for *in front of*) may select specific place concepts defined with respect to landmark objects. In this framework, place concepts model adpositions’ senses. Therefore, these senses are not unique, shaped by individuals’ views nor associated to place names.

Works within generative syntax built theoretical analyses on these insights. These works adopt “Place” as a label for a category projecting from spatial adpositions and case markers (e.g., *in*, *at*: Wunderlich, 1991, 1993; Koopman, 2000; Kayne, 2004). This category individuates concepts for regions defined via a landmark object (e.g., “in,” “front” regions). The emergence of the Syntactic Cartography framework introduces a refinement of this analysis (e.g., Cinque and Rizzi, 2010; Garzonio and Rossi, 2020). Cartography suggests that each morpheme in complex adpositions can project a distinct category. If we take *in front of* as an example, the morpheme *front* introduces a so-called Ax(ial)Part category, which introduces a “front” concept. The morpheme *in* projects the Place category, and the “place” concept associated to the “front” concept (Svenonius, 2006, 2008; Franco, 2016). The morpheme *of* projects a Kase category, and a relation between “front” location concept and landmark concept. We discuss the upshot of this account via (8).

(8) *The man sits in front of the table.*

According to this analysis, the PP *in front of the table* introduces a “Place” concept individuating a front location via a given table. If we take the use of this term as in geography and psychology, individuals may use PPs and sentences such as (8) for locations to which they have affective and social relations. Aside this being a problematic claim, it would entail that *in front of the table* could be treated as a place name for a generic location unoccupied by objects, i.e., not a “proper” place. Thus, these frameworks create wrong predictions regarding place concepts and their mapping to place names.

Formal, model-theoretic semantics offers a parallel set of theoretical issues when addressing the notion of “place.” For instance, Cresswell (1978) suggests that English adpositions (e.g., *over*) can denote “places” as possible destinations of moving entities, but does not define this notion. Model-theoretic formalisations of conceptual semantics propose that PLACE-functions denote unique regions in space, or *Eigenspaces*, via the location function ***loc’*** [i.e., we have ***loc’**(x) = l*]; Wunderlich, 1991, 1993; Zwarts and Verkuyl, 1994). Subsequent works propose that adpositions can denote regions or vectors (e.g., respectively, Nam, 1995; Aurnague et al., 1997; Krifka, 1998; Zwarts and Winter, 2000; Aurnague, 2004; Matushansky and Zwarts, 2019). Variants of DRT offer analyses of adpositions and spatial relations (e.g., Asher and Sablayrolles, 1995; Maillat, 2001, 2003; Roßdeutscher, 2013, 2014; Haselbach and Pitteroff, 2015; Haselbach, 2017). Thus, model-theoretic works address neither place Names nor place concepts.

Formal ontology works investigating linguistic issues present a different picture. Guizzardi (2005) suggests that adpositions can express mereo-topological (i.e., spatial “part-of”) relations (cf. also Egenhofer and Mark, 1995; Smith, 1995; Guarino, 1998). Smith and Mark (1998, 2001) suggest that generic terms in place names (e.g., *street*) may refer to “fiat” locations. Fiat (i.e., arbitrary) locations are such because humans can make complex assumptions on the properties/facets that individuate these locations (e.g., the postulation of boundaries: Smith and Varzi, 2000). These proposals treat place as a fiat type concept (Yue, 2013). They suggest that place names (e.g., *Sydney*, *Pitt Street*) encapsulate unique place concepts (e.g., “Sydney” as a unique city in Australia). Thus, these works analyse place names and adpositions as distinct categories realising distinct concept types within a rich ontology of “Space.” However, they do not explicitly state how one can analyse place names and concepts via these distinctions.

We can now summarise the picture emerging from Sections 2–4. Psychological models of place (e.g., Scannell and Gifford, 2010) converge on two assumptions. First, place concepts are shaped via the features that define places, though these features can be subjective and internalised as facets. Second, individuals and groups can internalise relations (e.g., forms of attachment) with these concepts. Crucially, facets theory and GIS models (e.g., Purves et al., 2019) suggest that place concepts and facets can be formalised via first order logic tools: geo-ontologies, representational tools (e.g., geo-atoms), and place networks. Third, linguistic frameworks do not lend themselves to integration in this picture. Some frameworks focus on pragmatic matters, thus explaining away the semantic properties of place names (Coates, 2017; Haspelmath, 2019). Other frameworks erroneously associate place concepts to adpositions (Jackendoff, 1983; Svenonius, 2010). Furthermore, ontology-driven and model-theoretical frameworks (Guizzardi, 2005; Haselbach, 2017) do not explore place names and their relations to place concepts. Therefore, (1)–(3), (6)–(8) and a unified model that makes correct predictions about place concepts, place names and their relations are still outstanding.

## 5. Place and place names: a unified account

The goal of this section is to outline a unified, multi-modal model of place names and place concepts that integrates different frameworks into a single theoretical architecture of cognition. Before we proceed, we clarify which version of DRT we implement and why.

DRT is a *dynamic*, *representational*, *modular* theory of meaning (Kamp et al., 2011: Ch. 1; Nouwen et al., 2016; Geurts et al. 2020). Dynamic, because it offers an incremental model of discourse interpretation that captures the role of extra-linguistic and linguistic context. Representational, because it manipulates *Discourse Representation Structures* (DRSs), which represent meanings as mental objects that speakers process as discourse unfolds. Modular, because it assumes that the interpretation algorithm takes structures (e.g., syntactic trees) as inputs, and returns DRSs as outputs. These structures can be connected with non-linguistic structures (e.g., concepts) via formally defined mappings, while being evaluated against a discourse context.

DRT comes in dozens of variants: many of these variants offer “layered” representations of linguistic content (e.g., Maier, 2006, 2015, 2017), and psychological states (e.g., Kamp, 2021, 2022). Crucial for our concerns is the fact that DRSs can be used as cognitive *Domain-general* Representation Structures.

Thus, via DRT, we can simultaneously model linguistic content, i.e., the senses of place names, and non-linguistic place concepts. In so doing, we can also integrate with DRT different theories on place (e.g., facets theories, formal GIS theories of place) that propose first order logic as a formal language. Therefore, we choose this framework as our starting point for two reasons. Theory-wise, DRT allows us to use one meta-language to connect different frameworks for studying place. Data-wise, it allows us to solve recalcitrant problems involving place names and place concepts via a multi-modal implementation of anaphoric relations.

We proceed as follows. We first introduce the basic principles of DRT as a theory of meaning (Section 5.1). We show how DRT permit us to formulate domain-general representations of meaning, and connect these representations via referential (“anchor”) relations (Section 5.2). We then move to the empirical applications, which cover place names’ grammar, semantic and lexical content, and their inter-subjective use in discourse (Section 5.3).

### 5.1. Discourse representation theory: key notions and a semantics for place names and place concepts

DRT can be conceived as a variant of first order logic enriched with relations among *discourse referents*. These are logical variables used to represent entities whose identity can be defined as discourse unravels (Kibrik, 2013: Ch. 2). Referents can be individuated via *conditions*: properties (i.e., 1-place functions) ascribed to referents, or relations (i.e., 2-place functions) holding between referents. The set of referents representing a discourse is known as the *universe of discourse* (e.g., *U = {x,y}*); the bundle of conditions, as the *condition set* (e.g., *S = [**cond***_***n***_***’**]*). A DRS is minimally formed by combining a universe and a condition set individuating and connecting these referents.

DRT has a well-known graphic format, or “box notation.” In this format, upper boxes represent universes of discourse as lists. Lower boxes represent condition sets as vertical lists, based on syntactic order of interpretation. Identity relations between referents act as *anaphoric* relations: pair-wise relations among different referents representing the same extra-linguistic entities. We illustrate these core notions via the example in (9).

(9) *A man walks in a park. He whistles.* [Adapted from Kamp et al., 2011: (16)]
(10) 

**Table 1:** 

x y z t **man’**(x) **walk’**(x) **in’**(x,t) t:**loc’**(y) **park’**(y) **whistle’**(z) **man’**(z) z = x

The two-sentence discourse in (9) establishes that there is a man walking in a park (first sentence), and that this man is also whistling (second sentence). The DRS in (10) introduces a possible representation of the sense assigned to this discourse. A set of conditions (e.g., ***man’***, ***in’***) defines the properties individuating each referent, and their possible relations. Referents have default existential import: for instance, *x* and *y* in the universe of discourse stand short for *∃x*, *∃y*. The sense assigned to the pronoun *he* introduces a new referent, identified as a man [i.e., ***man**’(z)*]. It then establishes that this referent is identical to the referent that *a man* introduces (i.e., *x*), via the anaphoric relation “z = x.” The two sentences form a coherent discourse, since they describe the same man [i.e., ***man’**(z) = **man’**(x)* also holds]. Each sentence also introduces other conditions individuating this man in discourse e.g., ***walk’**(x)* for the first sentence, ***whistle’**(z)* for the second sentence].

The DRS in (11) also shows that NPs referring to locations may introduce distinct referents and conditions. First, the condition ***park’**(y)* individuates a referent as a park. Second, a location function ***loc’*** maps this referent to its corresponding Eigenspace (unique location), represented via the referent *t* [i.e., *t:**loc**’(y)* holds: Wunderlich, 1991; Kamp et al., 2011: Ch. 4]. The condition/relation ***in’**(x,t)* holds between the location *t* as the landmark referent, and the walking, whistling man as another referent. The interpretation of conditions can be captured via Boolean conjunction (i.e., “⊓”). However, this assumption depends on DRT variants (Brasoveanu, 2007: Ch. 2; Brasoveanu, 2010; Brasoveanu and Dotlačil, 2020: Ch. 6). We will show that individuals may also interpret conditions via other schemas (e.g., Boolean disjunction “⊔”), depending in which context they access these representations. We expand this point and its empirical import as we proceed.

Our next step is introducing the two key assumptions by which DRT analyses proper names. First, proper names introduce the sets of conditions that uniquely individuate a referent: they act as “compressed” descriptions (Geurts, 1999; Maier, 2006, 2015, 2017; Kamp, 2015, 2021). Second, each name introduces a unique labelling condition as its sense. For instance, the condition ***Sydney’*** stands for a different set of conditions from ***Melbourne’***: they label distinct concepts for cities. Third, proper names and other parts of speech introduce different referent types: individuals (e.g., men), places (e.g., Sydney), and events, among others. Thus, an ontology for referents and conditions is also integral to DRT. We repeat (1) as (11) and offer its DRS in (12) to illustrate these assumptions.

(11) *The plane landed in Sydney.*
(12) 

**Table 2:** 

x^d, n = 1^ e^ev^ y^l,^ p^l, n = 1^ sy^d, n = 1^
**plane’**(x) e:**land’**(x,y) **in’**(y,p) p:**loc’**(sy) **Sydney’**(sy)

We discuss the conditions in (12) from bottom to top. The conditions *p:**loc’**(sy)* and ***Sydney’**(sy)* jointly read as follows. If *sy* is a referent representing Sydney as an individual/object (e.g., a physical agglomerate of buildings), then *p* is the unique location that this city occupies. Furthermore, *sy* is of type *d* (for individuals); *p* is of type *l* (for locations). The adposition *in* establishes that an internal location *y*, also of type *l*, is defined with respect to Sydney as a location Haselbach, 2017: Ch. 5; and references therein. The verb *landed* and the definite NP *the plane* establish that a plane (i.e., a referent *x* of type *d*) is in a landing relation with this internal location [i.e., *e:**land’**(x,y)*; ***plane’**(x)*]. The verb *landed* thus also introduces an event referent *e*. Once this verb combines with its syntactic arguments and their senses, it describes a “landing” type of event in which the plane and Sydney become “related.”^1^

The representation of referent types in DRT passes via superscripts in the universe of discourse (e.g., *x^d^*). Ontological distinctions can be modelled via hierarchical type theories (e.g., Asher and Lascarides, 2003; Asher and Pustejovsky, 2013; cf. also Asher, 2011). We however discuss such matters in Section 5.3.3, once we address Place concepts’ networks. At this stage, we require three types for our data: individuals *d*, locations *l*, and eventualities *ev*. Psychological and GIS works suggest that events play a role in the making of place (e.g., Scannell and Gifford, 2010; Purves et al., 2019; respectively). We show how this can be the case as we proceed. The uniqueness of referents can be represented via a super-script (i.e., *x^n = 1^*: cf. Kamp et al., 2011: Ch. 4; Kamp, 2015). Thus, the referents for the plane, Sydney and its corresponding place are unique, cf. (12).

The formal definitions for our discourse ontology are as follows. The set of spatial locations *L* does not overlap with that of individuals *D* and eventualities *EV*. The equation *D ⊓ L ⊓ EV = ∅* holds: the intersection(s) of the sets of referents belonging to these types are empty sets (i.e., individuals are neither locations nor events). The equation *D ⊔ L ⊔ EV=U* also holds: in our minimal ontology, the universe of discourse is made up of the union set of objects, locations and eventualities. We represent location referents *r* as *r^l^* (with *l ⊑ L*, “*⊑*” being the “sub-set/type of” relation: Carpenter, 1992: Ch. 2–3; Pustejovsky, 1995: Ch. 3; Asher, 2011: Ch. 2). We represent individual-type referents as *x^d^* (i.e., *x ⊑ D* holds); eventuality-type referents as *e^ev^* (i.e., *e ⊑ EV* holds). Event semantics approaches abound, and implement various sub-types of eventualities (Parsons, 1990; Landman, 2000; Rothstein, 2004; Ramchand, 2010). We address this matter as we proceed.

The status of *p* as a place referent representing Sydney thus emerges from three properties. First, *p* is a unique location: the city of Sydney as a unique object occupies this location (cf. individual referent *sy*^*d ,* *n = 1*^). Second, the sense of the place name *Sydney* establishes that a unique location *p^l^* defined via a unique set of conditions (i.e., ***Sydney’***) is under discussion. Place Names thus both label (i.e., describe) and refer to objects and the unique locations they occupy, i.e., places. Third, the unique place referent *p*^*l*,*n = 1*^ participates in two relations: a spatial relation with its internal location *y^l^*, and a landing relation with a unique plane *x*^*d ,n = 1*^. The landing relation, in turn, describes an event (referent) *e^ev^*.

We now have a model of the senses/meanings of place names. We can exploit DRT’s machinery to define place concepts as formal objects. Next, we reconstruct the notions of facet from psychology (Section 2) and geo-atom from GIS (Section 3) as DRT-based (i.e., logic-based) notions. In (13), we repeat the geo-atom for the Sydney Place concept from (5). In (14), we offer its DRS reformulation.

(13) *A:<Sydney, l, S = {extension, number-of-districts, popularity, citizens-opinion}, S(l(Sydney))>*
(14) 

**Table 3:** 

s^ο^ p’^λ^ S^ϕ^
p’:**l**(s) **S**(p’) = ⊔{**extension’**(p’) **number-of-** **districts’**(p’) **popularity’**(p’) **citizens-** **opinion’**(p’)} σ

The DRS representing the “Sydney” place concept includes an object referent *s* for the city. The localising function *l* geo-tagging objects is represented as ***l**(s)*. The set of facets individuating this place and their corresponding values are represented as conditions. For instance, the condition ***extension’**(p’)* represents Sydney’s spatial extension. The referents in this DRS belong to different types from discourse referents, because they represent our non-linguistic (e.g., visual) understanding of Place concepts. Though distinct, the corresponding ontologies are nevertheless inter-connected. We use Greek letters to represent these cognitive types: *ο* for objects (with *ο ⊑ Ω*), *λ* for locations (with *λ ⊑ Λ*), *ϕ* for (sums of) facets that individuate Place concepts (with *ϕ ⊑ Φ*). We assume that each type is disjointed from the other types/sets (i.e., *Ω ⊓ Λ ⊓ Φ = ∅* holds), and that the sum of these types forms the set of types from which geo-atoms are built (i.e., *Ω ⊔ Λ ⊔ Φ = Σ* holds). The sub-script *σ* represents the type of this DRS as a spatial (and platial) representation; for practical reasons, we insert it inside the DRS.

By reformulating geo-atoms/place concepts as cognitive types of DRS, we add one assumption about how facets can be represented. We have *S^ϕ^* as a referent representing the set of facets that individuate Sydney. Such referents are known as *structured referents*, in DRT (Asher, 1993; Asher and Lascarides, 2003; Brasoveanu, 2007; Kamp et al., 2011: Ch. 3–4). These referents can represent (union) sets of referents or sets of conditions. For instance, plural nouns can denote (union) sets of individuals [e.g., *the boys* may denote the set *A = ⊔{m,l} m*, *l* being two individuals]. Contexts can be modelled as sets of conditions (i.e., structured referents) that hold in discourse [e.g., *C(x) = ⊔{**cond***_***1***_***’**(x), **cond***_***2***_***’**(x)}* holds]. In our case, the identity ***S**(p’) = ⊔{**cond’***_***n***_*}(p’)* establishes that places can be individuated via a set of facets/conditions, descriptions of Place concepts that can become accessible in discourse.

Our novel re-formulation of place concepts as non-linguistic DRSs also offers two possible perspectives on facets. First, a unique combination of facets defines the conceptual content of a place concept: “Sydney” is shaped via a unique mental description or label (*S*, in (13)]. Second, the set union of these facets can be presented as a list of conditions [i.e., ***cond***_***1***_***’*** *⊔* ***cond***_***2***_***’**… ⊔* ***cond***_***n***_***’***): Sydney may be defined as beautiful, or an Australian city, or as a rugby haven, or also as a combination of all these facets. These two perspectives are equivalent: their role in DRSs and anaphoric relations is interchangeable, and thus equally connected to place names.

### 5.2. A multi-modal DRT architecture: connecting place concepts and place names

We now integrate different semantic (linguistic, conceptual) representations into one model of place names and place concepts. We choose *Grounded* DRT (e.g., Pross, 2010; Knott, 2012) for this purpose. In this variant, the flow of cognitive information is represented via three DRS layers: *Sensori-Motor Structures* (SMSs), *External Presentation Structures* (EPSs) and *Internal Representation Structures* (IRSs). SMSs represent perceptual information that individuals receive from objects in the world. EPSs represent mind-external objects and their relations mediated via perceptual information. IRSs build upon EMSs and SMSs and define constantly updating models of the multi-modal flow of information. Grounded DRT thus offers us a multi-modal, cognitively grounded model of how individuals can represent mind-external places as mind-internal concepts, and can use place names to discuss about these concepts.

**Figure 1:**
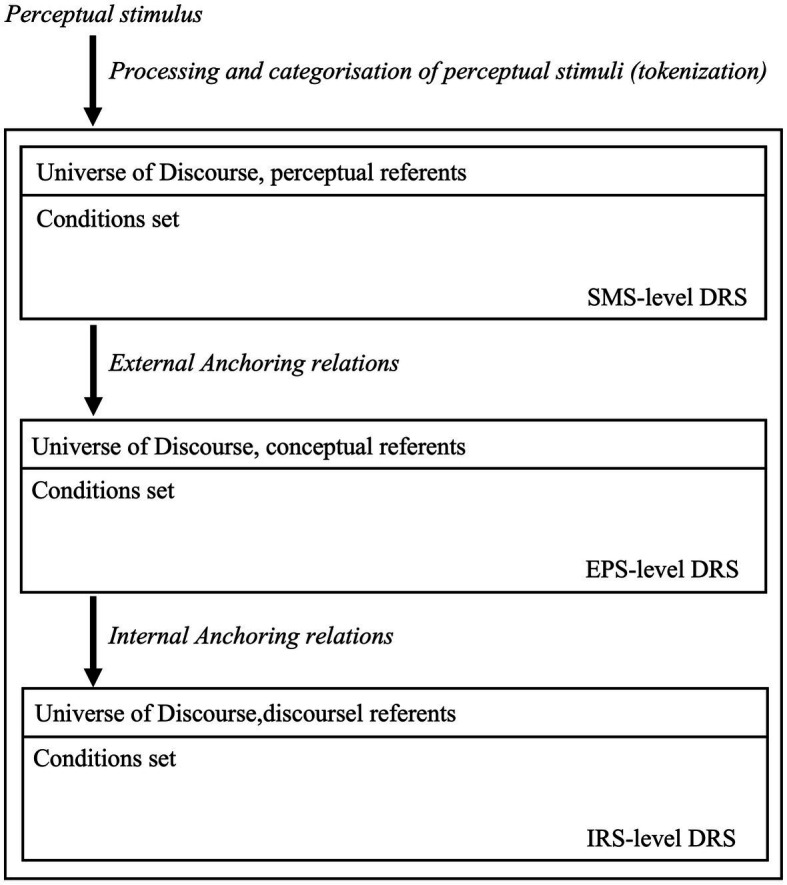
The model shows that SMSs are activated once perceptual information is received. The precise format of this information is not crucial, for our purposes (e.g., pictures vs. direct observation). Activation of an SMS licenses the activation of an EPS. Once the perceptual stream is processed, an individual has information about Sydney as a place; thus, the Sydney place concept is activated. The two layers become connected via anchors (see main text). The use of *Sydney* in sentences potentially describing this concept can then activate an IRS (i.e., a discourse-specific representation structure) and anchor it to perceptual and conceptual structures. Hence, from mind-external information about Sydney as a place, an individual activates the “Sydney” place concept, which can be mentioned in discourse via the place name *Sydney*. The model thus assumes that cognitive processes are modular and dynamic, but also inter-connected across layers of cognition, with perceptual representations acting as “grounding levels.”

We illustrate how this architecture works via a preliminary example. Imagine a context in which some individuals may decide to look up some information about Sydney. By consulting web-based content about this city, the individuals may temporarily access the “Sydney” place concept that the individual has memorised over time. The multi-modal representation in Figure 1 represents this information flow.

As the model shows, a directed flow of information connects the different DRS types. This flow is regulated via mappings known as *anchoring relations*, or anchors (Kamp et al., 2011; Ch. 6). Anchors are relations between referents belonging to different layers. They represent how the mind integrates representations across levels of comprehension by connecting their corresponding referents. Anaphoric relations are a sub-type of anchors in which referents belong to the same type and layer (e.g., *x^t^ = y^t^* minimally holds). General anchors, instead, include functions mapping one referent from one layer, to a referent of another layer [i.e., *f(x^τ^) = y^t^* minimally holds; Ursini, 2011; Ursini and Acquaviva, 2019]. Anchors involve initial or “source” referents, and final or “floater” referents. We introduce the relevant definitions in (15) (cf. Pross, 2010, p. 41, definition 7).

(15) a. *Given referents x, y, an anchoring relation is the duple ANCHOR < x,y>, with x a source and y a floater;*
    b. *An anchor is:*
    • *internal if its floater is an IRS-reference marker and its source an EPS-reference marker;*
    • *external if its floater is an EPS-reference marker and its source an SMS object term;*
    • *anaphoric if its floater is an IRS-reference marker and its source an IRS-reference marker*.

Crucially, if we assume that anchors connect referents across different layers, we predict that these relations can also include structured referents. Place concepts can introduce sets of facets as descriptions (e.g., ***F***), which can then become part of anchors. The condition(s) ***name’*** that place names carry become the internal (i.e., language-based) floaters of place concepts. This prediction can be made precise as follows. Consider a context in which we observe a touristic advertisement on-line, showing a plane landing in Sydney’s main airport. In this context, we can use (11) to minimally describe the event we perceive, and the “Sydney” place concept we entertain while watching this video. In this context, we also develop the most minimal form of cognitive attachment towards this concept: we acknowledge its existence in our mental life.

We next introduce a representational format for relations between concepts and individuals. In DRT, individuals entertaining representations can be represented via an indexing mechanism (Kamp et al., 2011: Ch. 7; Maier, 2015). If *A*, *B* represent individuals in discourse exchanging information, then *A:DRS* and *B:DRS* represent relations between these individuals and distinct DRSs that these individuals can entertain. We can now represent place attachment relations in their minimal form. *A:P_DRS_* can be taken as a shortcut for *R < A:P_DRS_>*: an individual *A* is attached to a concept for a Place *P*, represented as a DRS, via a relation *R*. For instance, *love < A:Sydney_DRS_>* is an attachment relation in which an individual *A* loves “Sydney” as a place concept. More in general, attachment relations represent the fact that individuals relate themselves to the place concepts they can use and exchange in any mental and social activity.

**Figure 2:**
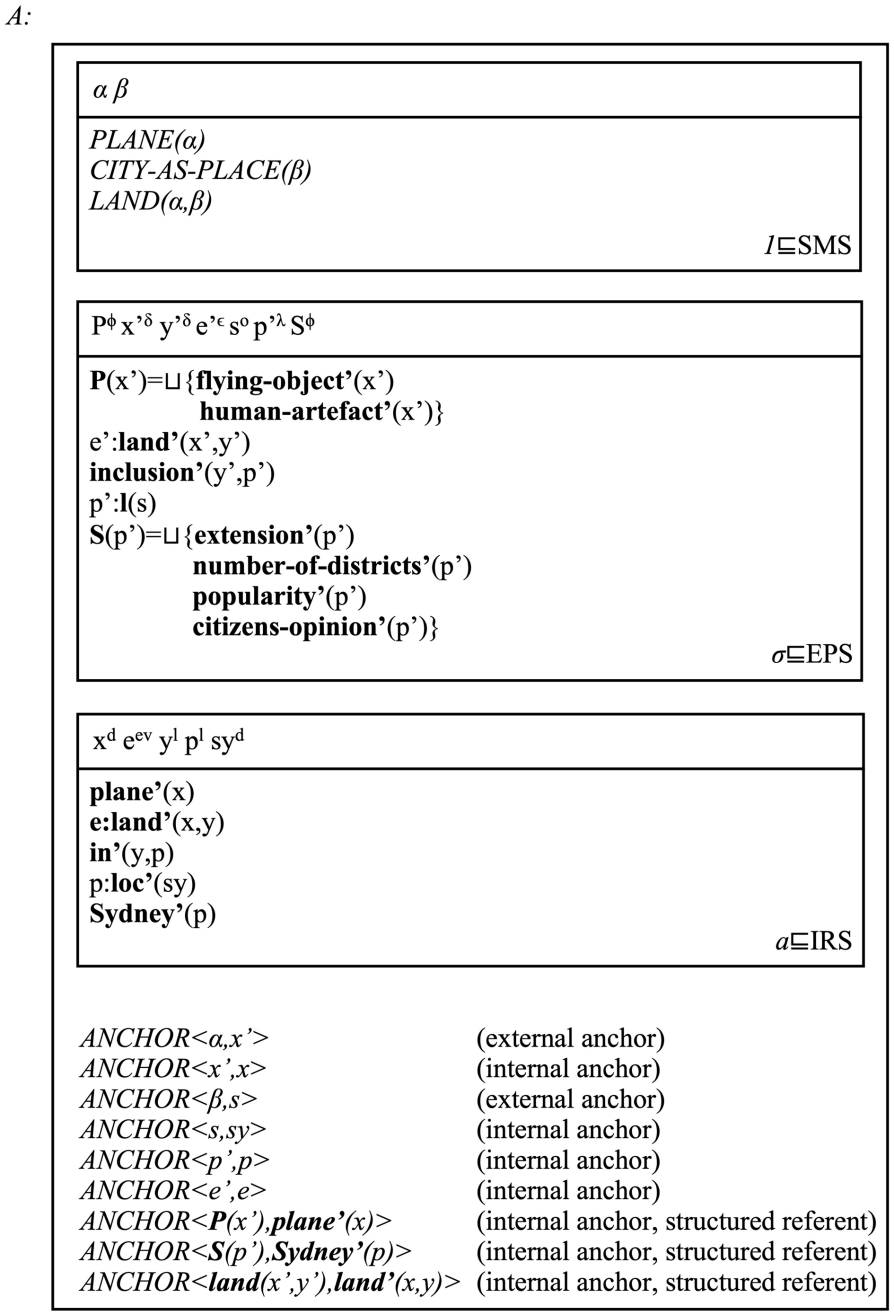
A multi-modal DRS for the sentence *the plane landed in Sydney* and its extra-linguistic context of interpretation, and as entertained by an individual *A*. We follow Pross (2010) in using Greek alphabet letters to represent EPS referents, and capital letters for the conditions individuating these referents. No ambiguity with types should arise, given the multi-modal/layered format of representations. Conditions in SMS-level structures display perceptual information allowing individuals to individuate entities and their possible relations (e.g., one referent looking like a plane, one looking like a city, the landing relation). We also follow Pross (2010) and use undecomposed conditions written in capital letters (e.g., *PLANE*) to represent this perceptual information in a compact manner. This DRS represents a distinct entity belonging to the SMS type (i.e., the relation “1 ⊑ SMS” holds). The condition *CITY-AS-PLACE* represents how Sydney is perceived as a place, i.e., a mind-external (complex) object occupying a location. The DRS belonging to the conceptual (i.e., EPS) layer includes the “Sydney” place concept in its extended form, but the “plane” and “land” concepts in their compressed forms. We focus only on this Place concept to better explain how anchors work. We represent place concepts as sets of facets identified with a structured referent: ***P*** is the set of facets identifying a plane concept, and ***S*** is the set of facets identifying the “Sydney” concept. We do not attempt to be exhaustive with our lists: their purpose is to hint at the complexity of concept representations, as we fully discuss in Section 5.3. We represent the fact that the DRSs belonging to the type *σ* of spatial (re)presentations are in turn part of the EPS (i.e., conceptual) layer via the relation *σ* ⊑ EPS. The relation *a* ⊑ IRS represents the fact that DRSs belonging to the type *a* (i.e., DRSs for assertions) are in turn part of the IRS layer (i.e., the discourse level). The Supplementary file offers further observations about anchors and ontological matters.

Figure 2 shows how our model integrates all this information.

The place name *Sydney* refers to the place concept “Sydney,” which in turn represents individual *A’*s perception of Sydney as a mind-external place. This reference act passes via three anchors. Thus, *ANCHOR < **S**(p’),**Sydney’**(p) >* establishes that ***Sydney’***, the sense assigned to this place name, refers to (i.e., labels) the unique facets set individuating the “Sydney” concept. *ANCHOR <β,s>* and *ANCHOR <s,sy>* establish that the tokens of these senses and concepts (i.e., the referents *sy^d^*, *x^δ^* and *α*) are also connected. An individual *A* can see a video of the city of Sydney and activate a visual and conceptual referent to mentally (re)present it. The individual can then use the place name *Sydney* to refer to the corresponding place concept, “Sydney.” Place concept and place name can be anchored to the places that these facets describe. However, perceptual referents and SMSs must represent these entities, i.e., connect perceptual information to mind-external entities such as places, planes and so on.

One consequence of this result is that we must include eventualities as part of our ontology of Place concepts. We include the set *Ε* of eventualities in our Place ontology: *Φ ⊔ Λ ⊔ Ε = Σ* holds (Casati and Achille Varzi, 2015; Guarino and Guizzardi, 2016; Guarino et al., 2022). This formalisation captures the assumption in psychology (e.g., Seamon, 2012; Scannell and Gifford, 2017) and GIS (Purves et al., 2019) that place concepts are also defined via the events that can happen in them. Furthermore, it is consistent with recent research showing that events are distinct concept types (Zacks and Tversky, 2001; Zacks et al., 2007; Zacks and Swallow, 2007; Zacks, 2014). Our conceptual ontology for place only lacks a type for facets to reach full psychological plausibility; we defer their introduction to Section 5.3.3.

The model we have defined so far can be interpreted as follows. An individual can see images of the city of Sydney and read sentences such as (11). When this happens, the various perceptual stimuli (images, text) activate the individual’s place concept of “Sydney” via its multiple facets. The individual then connects this concept to the place name *Sydney* and the sentence in which this name occurs. The multi-modal Domain-general Representation Structure (DRS) in Figure 2 represents these interwoven facets by which this individual makes sense of this place in context. We must now integrate this semantic model of place names and place concepts with a novel morpho-syntactic analysis of place names.

### 5.3. The proposal: core applications and results

The goal of this section is to present the model’s empirical import. To maintain our discussion focused but empirically sound, we offer a multi-modal account of place names with discourse-bound applications. We start by connecting place concepts and place names (Section 5.3.1). We then offer an account of adpositions that establishes their differences with place names (Section 5.3.2). We integrate the model with a frames theory analysis of conceptual and lexical relations, thereby defining a minimal model of place conceptual networks (Section 5.3.3). We conclude by showing how can we model place attachment relations in discourse-oriented, inter-subjective models in context (5.3.3–5.3.4). From here onwards, we omit uniqueness superscripts in DRSs unless necessary, to increase readability.

#### 5.3.1. A model of place names

We begin with a novel account of the grammatical structure of place names.^2^ We treat place names as coordinated phrasal compounds, as standardly assumed for (complex) proper names (Jackendoff, 2009; Koptjevskaja-Tamm, 2009, 2013; Schlücker and Ackermann, 2017; Schlücker, 2018, 2020). The phrasal status of generic terms is justified because they can also occur as common nouns (e.g., *street* in *the street is wide*) or as adjectives or other categories (e.g., *blue* in *Blue Mountains*). Specific terms are either proper names or bare common nouns, i.e., NPs (e.g., *Pitt* in *Pitt Street*; *Mountains* in *Blue Mountains*). A potentially silent head connects the two phrases, although in place names such as *Isle of Wight*, *of* can project this head. We suggest that this head is a type of “relator” connecting two NP-like elements, and thus it projects an N head (den Dikken, 2006: Ch. 4; Bauer, 2009; Scalise and Bisetto, 2009; Köhnlein, 2015).

**Figure 3:**
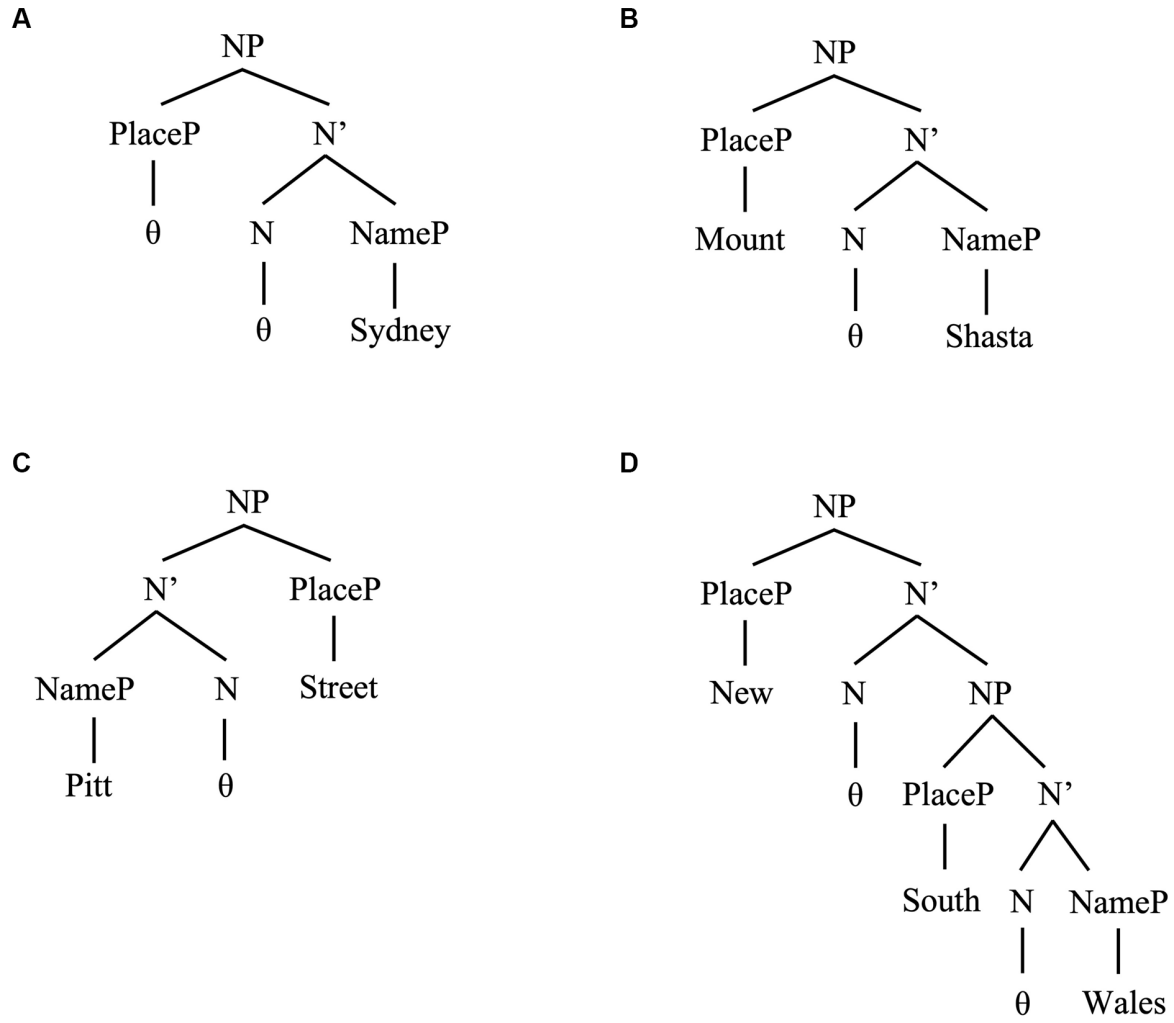
We associate the structure in **(A)** to *Sydney* and other place Names only including a “bare” NameP. For *Mount Shasta* and similar other names, we assume the structure in **(B)**. The mirror structure in **(C)** can represent place names such as *Pitt Street*. We also assume that the two mirror orders are base-generated, i.e., that PlacePs and NamePs can be linearized on either “side” of an N head (cf. David, 2011; Blair and Tent, 2015, 2021; Ursini, 2016). In either case, NamePs are complement phrases of N; PlaceP, specifier phrases of this head. For place names such as *New South Wales*, we allow recursion of PlacePs, as **(D)** shows. Note that we treat *New* and *South* as projecting PlacePs: they classify the type of place concept they name according to residing hemisphere (i.e., *South*) and historical novelty (i.e., *New*). The Supplementary file contains more observations on these structures.

We assume that recursive structures are also possible (cf. Romanova and Spiridonov, 2018). Place names such as *New South Wales* can include two generic terms, *New* and *South*, and the specific term *Wales*. They can also introduce multiple silent heads connecting these terms into recursively built NPs. Place names may only include specific terms and thus amount to bare NPs, e.g., *Sydney.* We illustrate the proposed structures in Figure 3.

We use the categorial label PlaceP to underline that generic terms individuate place concepts such as mountains, cities and streets. Some generative works suggest that the category “Place” can act as a sub-type of a Classifier Phrase (e.g., Kayne, 2004; Romanova and Spiridonov, 2018). Specific terms, instead, name individuals (e.g., families, *Pitt*; cities as constructs, *Sydney*). We use the category NameP to capture this fact, treating it as a sub-type of (bare) NP (Anderson, 2004, 2007; Van Langendonck, 2007). The term “place name” thus appears as a slight misnomer. The term however encapsulates the fact that categories referring to place concepts and individual concepts combine to form place names.

Before we continue, note that we assume that bare place names, e.g., *Sydney*, may involve the projection of empty categories [i.e., no term projects PlaceP in (a)]. However, it is generally assumed that bare place names such as *Sydney* are semantically equivalent to formal counterparts including generic terms, such as *city of Sydney* (Blair and Tent, 2015, 2021; Perono Cacciafoco and Cavallaro, 2023: Ch. 2). In our account, both forms have the same syntactic structure, but *Sydney* corresponds as a reduced form of the more formal place name *City of Sydney*. Semantic equivalence holds because of this relation.

We can now anchor this grammatical account to a semantics for Place Names, in turn anchoring this content to place concepts. We assume that NamePs can introduce reference to any entities from which a place may receive its name (e.g., a wealthy family for *Pitt*). The relator N introduces a relation holding between these referents (Matushansky, 2009). We propose that this relation implicitly describes a “baptism” or naming event (Coates, 2017; Blair and Tent, 2021). We suggest that all categories denoting relations (here, N) introduce event referents that these relations describe (Landman, 2000: Ch. 8; Ramchand, 2010). We thus represent the fact that individuals may tacitly assume that a place received a name in a socially sanctioned event. The nature of this event, i.e., its etymological roots, may remain unknown to them. Nevertheless, it guides their interpretation of a place name’s sense by also establishing that a community, at some stage, bestowed this name to the place to which the name refers. Knowing the meaning of a place name thus also means knowing the existence of this baptism event.

The next step is to anchor place names as linguistic (i.e., grammatical, semantic) units to place concepts as non-linguistic units. We standardly assume that reference is defined at a phrasal level (e.g., Kamp et al., 2011: Ch. 2; Coates, 2017; Acquaviva, 2019; and references therein). We thus predict that reference is a “layered” property. Anchors among referents entail anchors among their facets. In our case, anchors among referents for place concepts and place names entail anchors between the facets and the linguistic meanings shaping these distinct units. That is, the meaning of *mount* in *Mount Shasta* refers to the bundle of facets defining a “mount” place concept.

**Figure 4:**
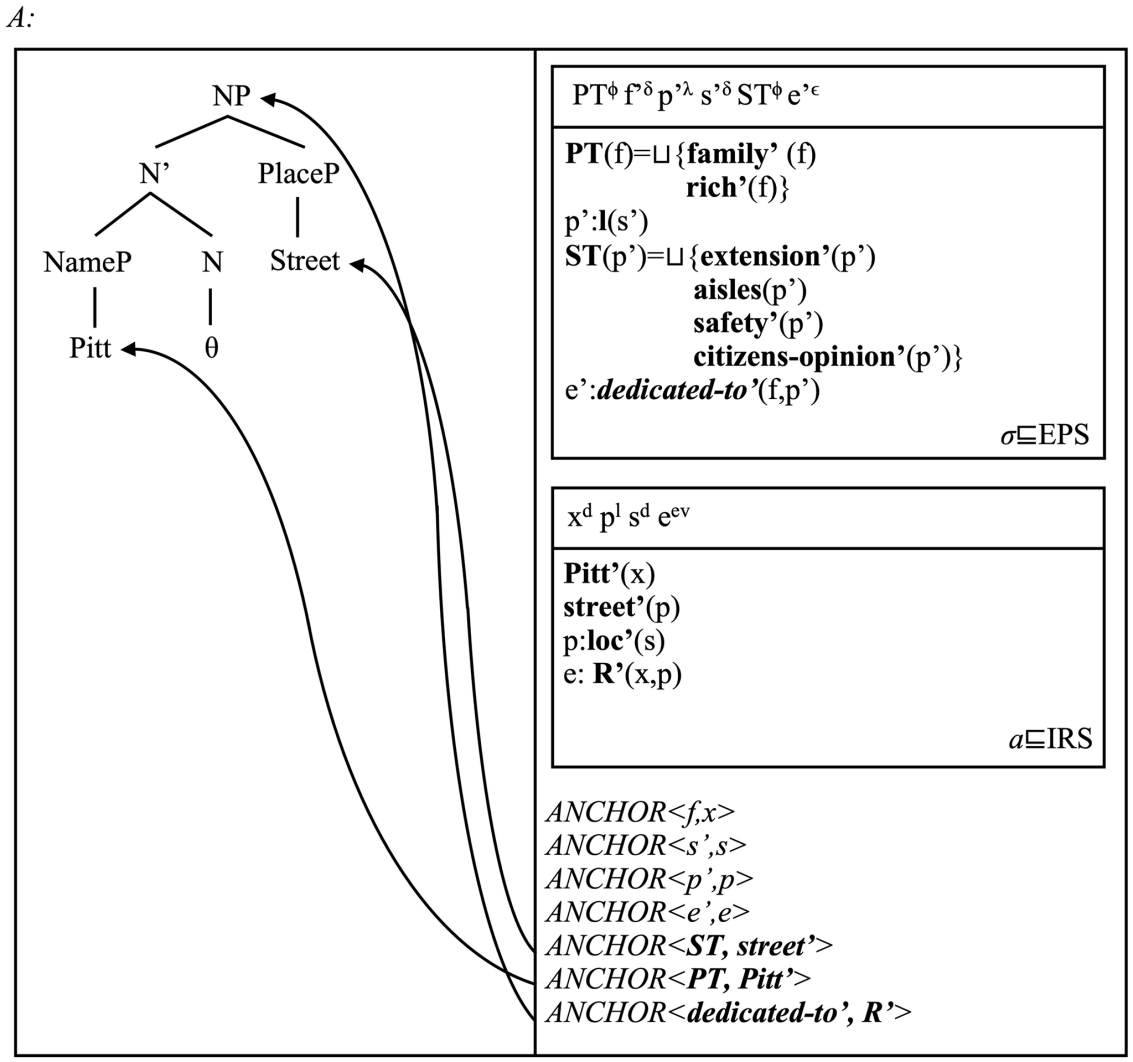
This multi-modal representation reads as follows. The spatial DRS (typed *σ*) includes ***PT*** as the set of facets individuating a concept for the Pitt family. The set ***ST*** includes the facets individuating a street place concept. The relation ***dedicated-to’*** represents the possible conceptual link between family and street concepts. Speakers may not know of the etymological roots of this Place name, but at least know that some conventionally defined naming relation holds between these concepts (cf. Coates, 2017; Perono Cacciafoco and Cavallaro, 2023: Ch. 3). There is strong evidence that naming relations come into different types and define conceptual hierarchies (e.g., Blair and Tent, 2015, 2021; Tent and Blair, 2011). Here we use ***dedicated-to’*** as a partially descriptive label, so that readers may easily remember the role of this class of relations. The event referent *e’*, belonging to the conceptual type *ϵ*, therefore represents the naming event establishing this relation within a linguistic community. From here onwards, we omit SMS-type DRSs representing perceptual information for clarity. The Supplementary file contains more observations.

**Figure 5:**
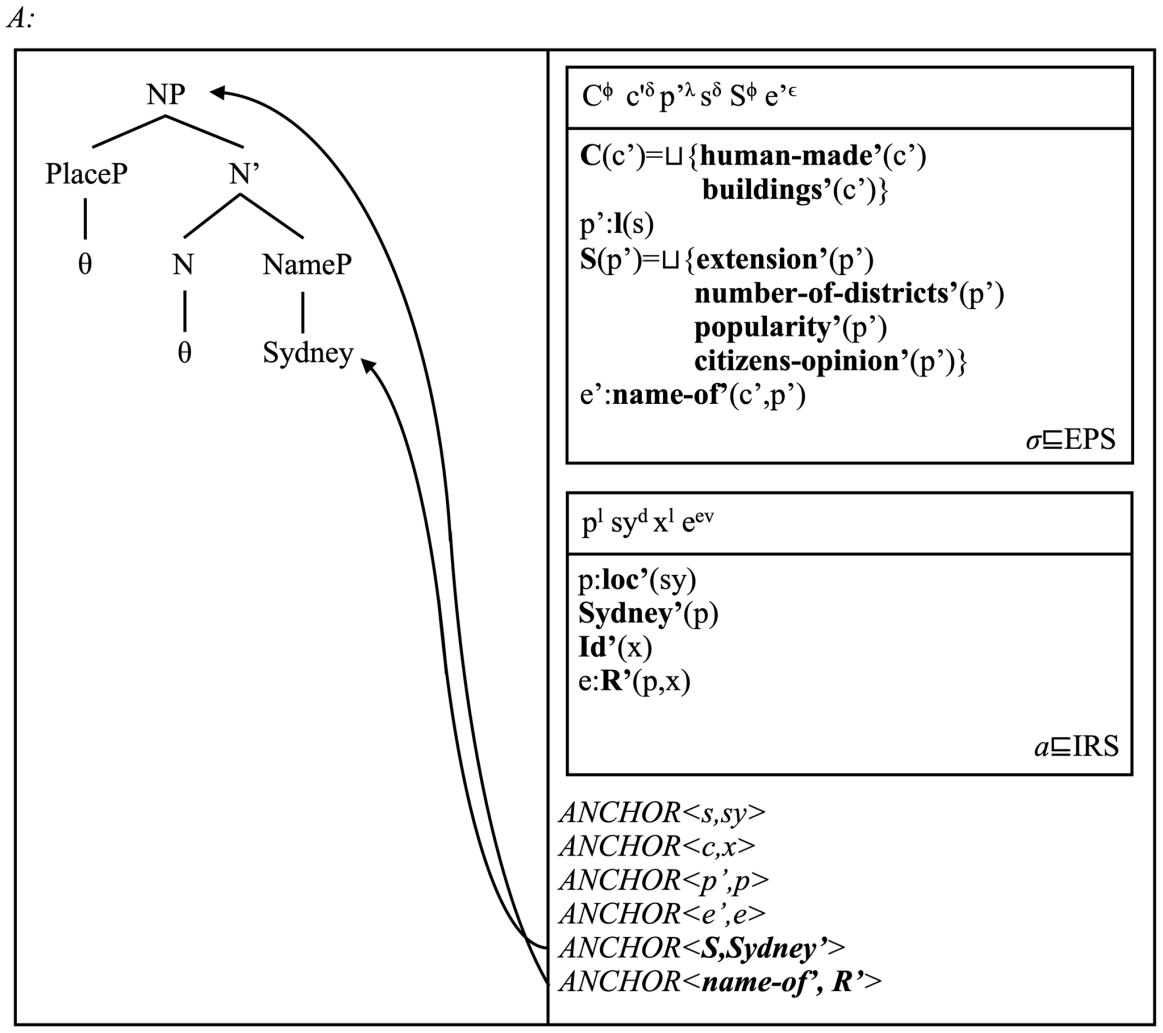
This representation of *Sydney* and the “Sydney” concepts acts as a (final) extension to our analysis, and reads as follows. The DRS *σ* includes information about the specific place type for Sydney: a city, defined via its set of (non-exhaustive) facets ***C***. The possible relation between these facets and those individuating the specific place (i.e., ***S***) can be a “simple” naming relation (i.e., ***name-of’***). Crucially, the lack of an overtly realised PlaceP term in the place name entails that the “city” concept remains unexpressed: no explicit label refers to this concept, i.e., ***C*** remains unanchored. Conversely, a representation for the formal name *City of Sydney* would include an explicit anchor to the ***C*** concept. The Supplementary file contains some more observations.

We also predict that place names introduce three “points” of reference to conceptual structure. First, NamePs refer to the entities bestowing names to places, via the concepts associated to them (Kamp, 2015, 2022; Maier, 2017). Second, PlacePs refer to the (structured) facets describing place concepts. Third, place names as “whole” NPs refer to naming event connecting naming entity and named Place. We show the import of all these assumptions via the analysis of *Pitt Street* in Figure 4, and *Sydney* in Figure 5.

#### 5.3.2. Place concepts and networks: the case of adpositions

As discussed in Sections 2–3, psychology and GIS models suggest that Place concepts can enter different types of relations, thus forming “place networks” (e.g., Scannell and Gifford, 2010; Purves et al., 2019). The sentences in (1)–(3), (6)–(8) offer evidence supporting this claim. Individuals who use these sentences in discourse can describe spatial relations between *Sydney* and *Pitt Street*, and between *Sydney* and *Brisbane*. They can also express how they internalise the place concepts associated to these cities and streets. Our conceptual knowledge of places can include facets defining these places and the relations holding among these places: place networks. These relations are expressed via verbs and adpositions in discourse; thus, we need a minimal linguistic model of verbs and adpositions to account for place networks.

**Figure 6:**
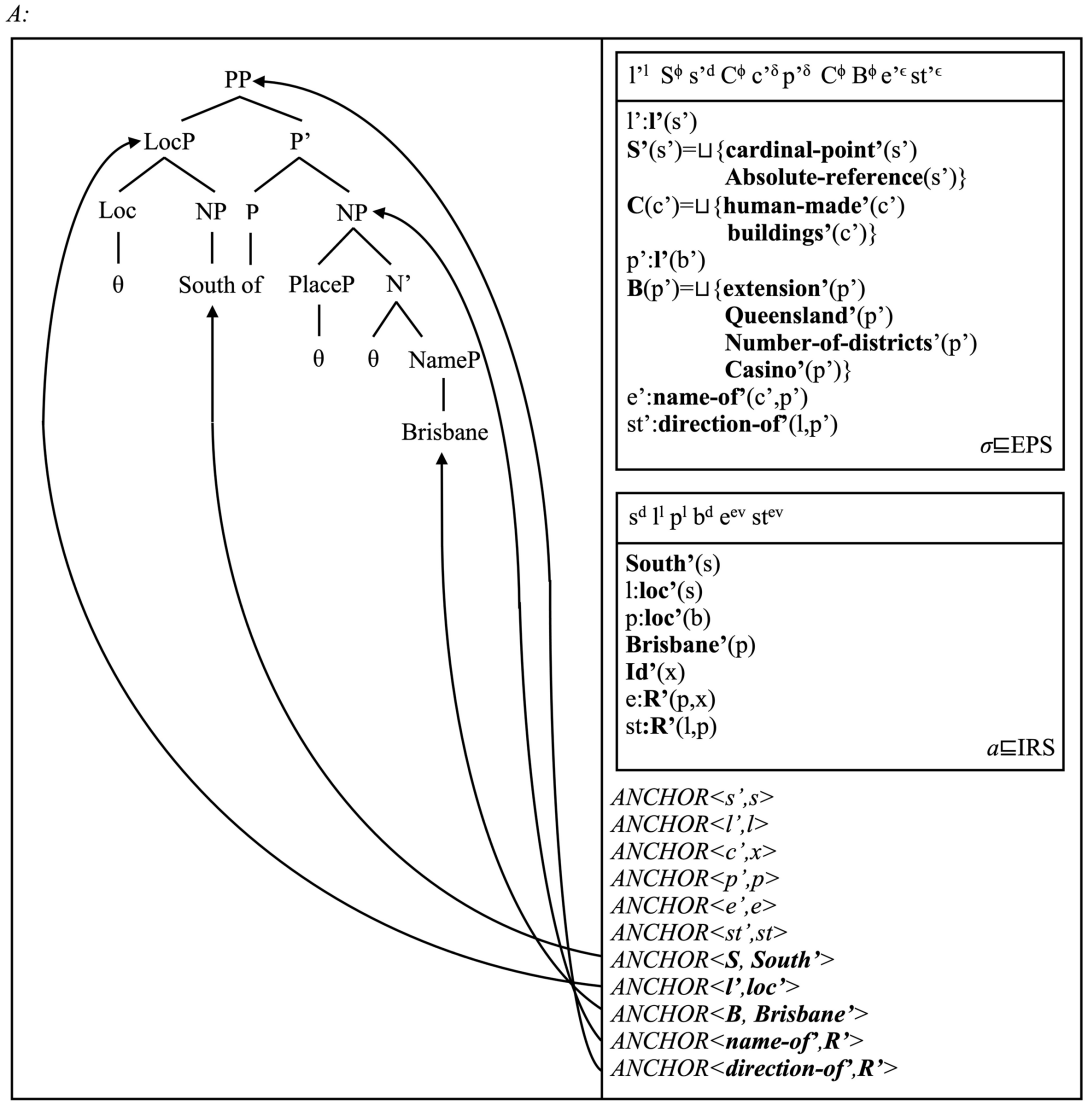
The PP *South of Brisbane*, from the sentence *Sydney is South of Brisbane* [i.e., (8)], is assigned the structure on the left, and the multi-modal DRS on the right. The structure shows that adpositions and the PPs they form include five potential sites for the introduction of anchors: the ground NP (here, *Brisbane*), the specifier PP (i.e., *South* forming a LocP with a silent head), and the PP as a complex phrase. A fourth site is the relational NP (i.e., *South*, *front*) as an NP embedded in the adpositional domain. The Supplementary file contains more observations. Note that we remove the individual’s index to increase readability.

**Figure 7:**
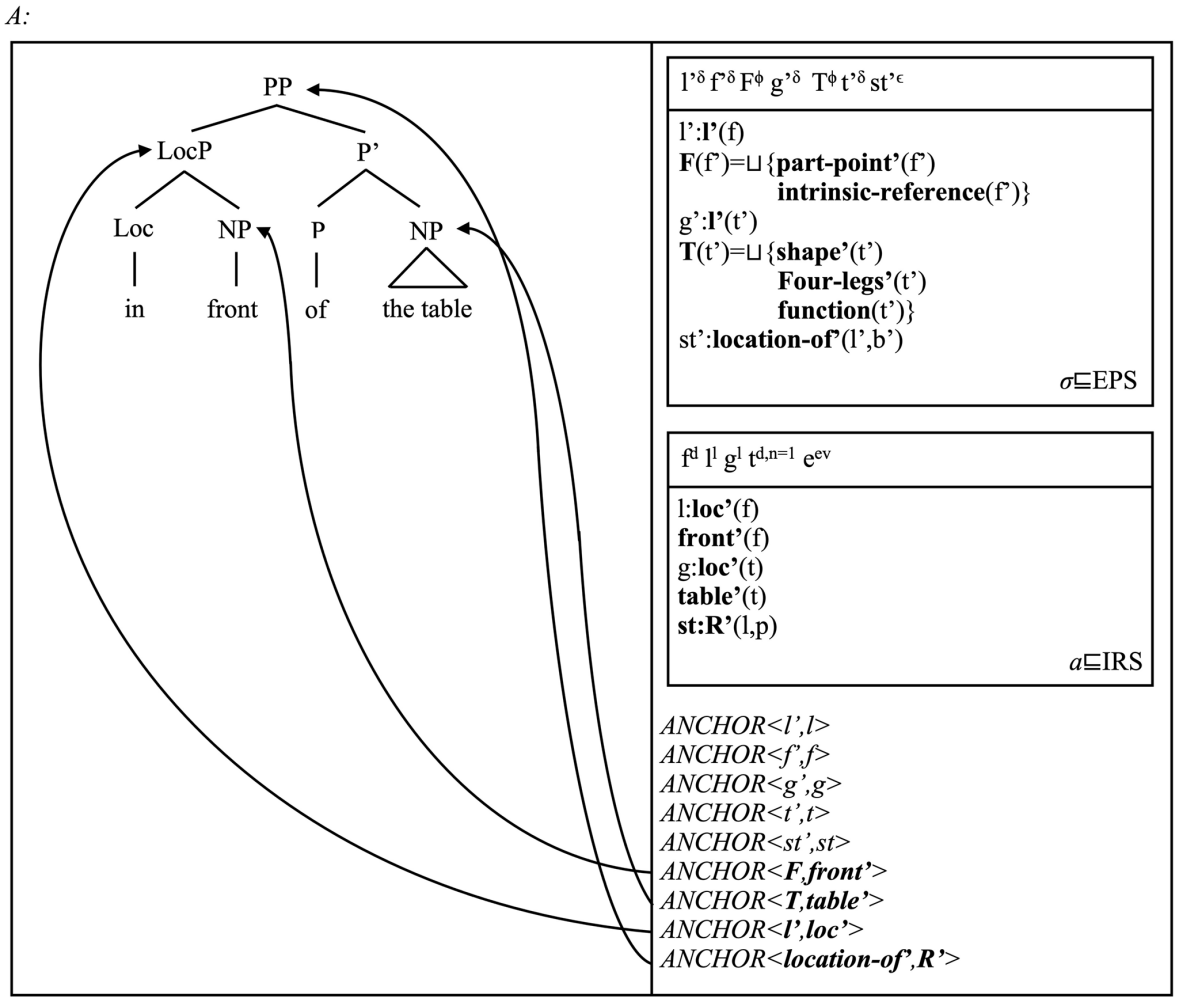
The PP *in front of the table*, from the sentence *the man sits in front of the table* [i.e., (8)], is assigned the structure on the left, and the DRS on the right. The analysis confirms that adpositions introduce reference to location and reference system concepts via their embedded nominal elements (here, *front*). The analysis also shows that definite NPs such as *the table* may introduce unique referents in discourse, as we mark in the universe of the discourse DRS (i.e., *t^d,n = 1^*). This uniqueness condition can determine the uniqueness of the location that this object occupies, in a manner similar to how place concepts involve unique objects and their locations. However, unlike place names, this NP does not introduce a naming event for this table. This is the case because NPs other than proper names introduce referents instantiating these concepts via different grammatical and conceptual mechanisms, such as discourse marking [e.g., (in)definiteness], but also via quantification [e.g., the indefinite *a park* in (3)]. In other words, while spatial adpositions can introduce relations among locations and places, place names introduce reference to one selected place. Our account correctly captures and predicts this division of labour.

We begin by offering an alternative analysis of adpositions to those discussed in Section 4 (cf. Emonds, 1985; Acedo-Matellán, 2016; Matushansky and Zwarts, 2019; Ursini, 2020; Ursini and Tse, 2021). We assume that complex adpositions (e.g., *in front of* or *South of*) include a relational noun/NP (*front*, *South*). These NPs may form a phrase with a possibly silent first adposition [e.g., *in* and *front* form *in front*; *South* and “∅” form the phrase *(∅ South)*]. We assume that the first adposition projects a “Loc(ative)” head: it maps the object that the NP denotes onto a location. The resulting LocP(hrase) enters a relation with this NP (e.g., *the house*) via a relational adposition P (e.g., *of* in *South of*, *in front of*). The PP introduces a relation between two spatial concepts: one for the landmark object referent, one for a second location defined via this object. Our model thus predicts that LocPs, i.e., landmark and relational NPs, PPs introduce reference to matching spatial concepts. We show these predictions via the pair of PPs *South of Brisbane* and *in front of the car* in Figures 6, 7.

As the DRSs in Figures 6, 7 show, relational NPs can introduce reference to object concepts such as (abstract) cardinal points (*South*) or parts of grounds (*front*; Tenbrink, 2011; Rybka, 2015). Once they merge with Loc heads, a LocP introduces reference to a location concept and the type of spatial reference system they represent. Thus, the “bare” NP *South* introduces reference to a location that is part of the polar coordinates system. *In front* can instead introduce reference to a location defined via the parts of an object. Once full PPs are formed via the head *of* and a landmark NP, the PPs introduce spatial relations defined via these reference systems. This view is consistent with standard views on the semantics of adpositions and reference systems (e.g., Levinson, 2003: Ch. 1; Levinson and Wilkins, 2006: Ch. 1; Cablitz, 2006; Palmer, 2015).

**Figure 8:**
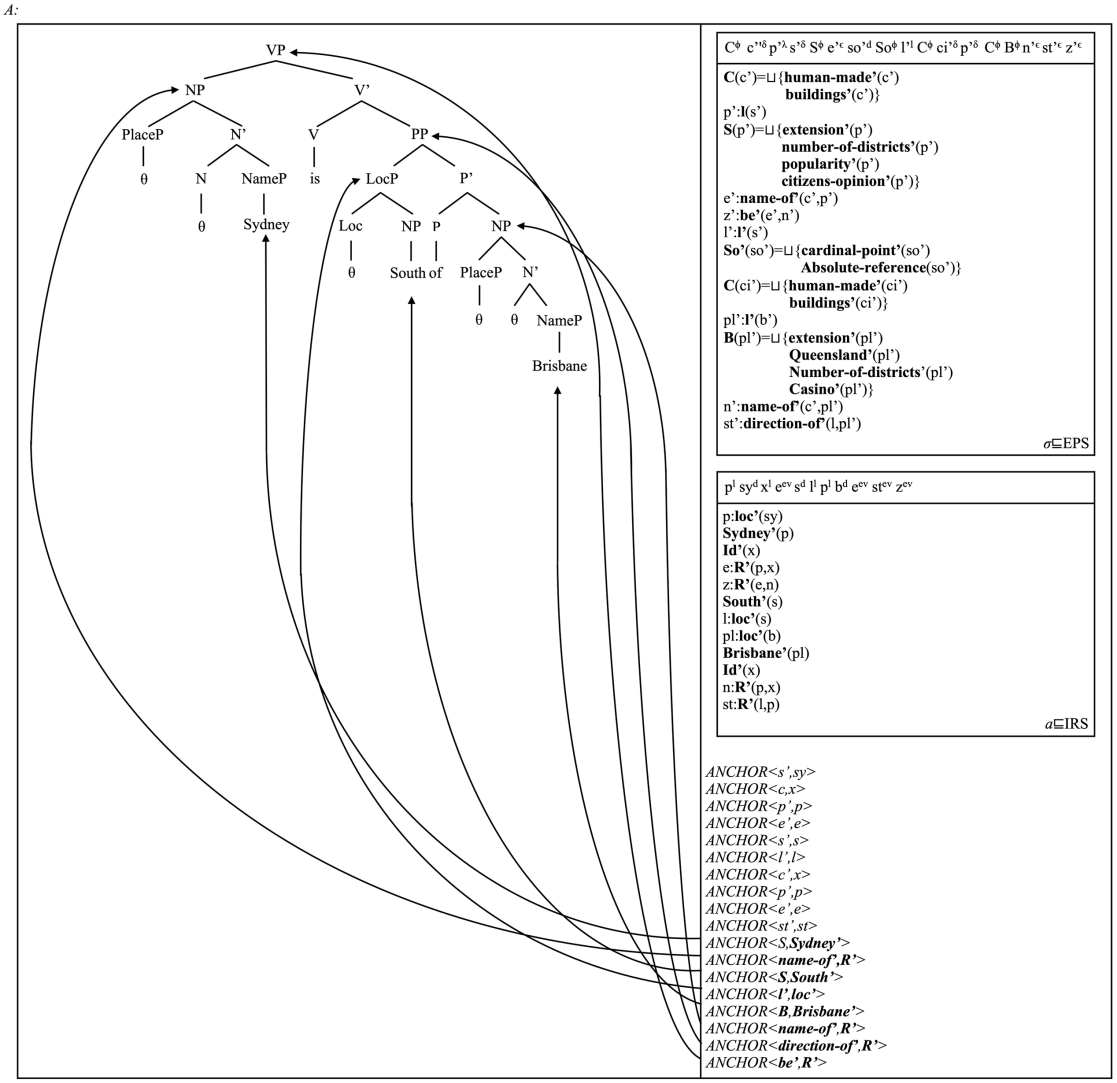
The sentence in (6), *Sydney is South of Brisbane*, is assigned the structure to the left and the DRS to the right. All anchors and conditions correspond to those discussed in Figures 5, 7; hence, we trust the readers to find their role clear. We assume that the copula *is* acts as a simple relational head establishing a form of identity between referents (cf. den Dikken, 2006: Ch. 3; and references therein). The anchor mapping the conceptual relation holding between “Sydney” and “Brisbane” as place concepts is *ANCHOR <**be’**, **R’**>*, i.e., the anchor establishing that a sentence (i.e., a bare-bones VP) introduces this relation and reference to the *z* state it represents. The conceptual relation between the “Sydney” and “Brisbane” places is expressed via this predicative sentence. It establishes that the first place, “Sydney,” lies in the (broadly) defined South region of a second place, ‘Brisbane.”

We can then show how spatial adpositions and verbs can define relations over place concepts (cf. Figure 8). To capture this fact, we offer a minimal structure for the sentence in (5). We assume that the copula projects a verb head V, and forms a “bare-bones” VP as a minimal clause (cf. Figure 8). As the layered DRS shows, *Sydney* and *Brisbane* refer to the Place concepts for these two Australian cities. Instead, *South of* introduces reference to a “South-of” relational concept. Other spatial relations can certainly be defined, via the content of their relational NPs (e.g., ‘inclusion’, ‘part of’: Aurnague et al., 1997; Zwarts and Winter, 2000; Haselbach, 2017: Ch. 5).

Crucially, this analysis correctly distinguishes the properties of place names from those of other NP types. For instance, we predict that the indefinite *a park* in (3), the definite *the table* in (8) do not refer to place concepts. The indefinite NP does not refer to a unique location (i.e., there can be more than one park); the definite NP refers to a unique location, but one lacking a name that establishes its status as a place. Our model can therefore account for why the place names *Sydney* and *Pitt Street* in (1)–(2) introduce reference to Places and their respective concepts, and why the PP *in a park* in (3) refers to a general location.

Thanks to this result, our model can also solve the theoretical problems arising in conceptual semantics and generative approaches (again, Jackendoff, 2002; Svenonius, 2010). More importantly, this view highlights the linguistic and conceptual differences between place names and adpositions (here, adpositions). Adpositions refer to locations and spatial reference systems via the category LocP: they introduce spatial relations. Place names are instead anchored to place concepts as possible arguments of spatial relations. The two categories thus have different roles and properties within grammar. This fact can be captured only by formally distinguishing the functional and lexical heads forming these categories (e.g., Loc for adpositions, Place for place names).

### 5.3.3. Conceptual place networks: a lexicon of place names

We can now define place concepts networks. For this purpose, however, we must extend our approach to a formal theory of hierarchies.

Various disciplines within cognitive sciences use *frames* as a representation format for any concepts (Barsalou, 1992a,b, 1999; Gamerschlag et al., 2014: Ch. 1; Gamerschlag et al., 2015). Frames can be recursively defined as sets of attributes and the values associated to these attributes. For instance, the concept MAMMAL can be defined via the attribute *animal*, which can have further attributes as its values: *bear*, *human* and several others. The attribute *human* can have more specific attributes as values (e.g., *woman*, *child*). Attributes can be classificatory or relational: they classify entities described via concepts, or establish relations among entities and/or concepts (e.g., Petersen, 2007; Naumann, 2013). The framework known as “Frame Semantics” models word senses via frames (Fillmore, 1982; Fellbaum, 1998; Fillmore et al., 2003; Fillmore and Baker, 2010; Busse, 2012). Word senses can involve reference to different attributes individuating concepts. As words can share attributes and/or values they encapsulate, they can also enter hierarchical (or “inheritance”) relations reflecting these relations among attributes. For instance, the attribute *child* is a sub-type of *human* attribute, which in turn is a sub-type of *animal* attribute.

Recall now that models of Place attachment (e.g., Scannell and Gifford, 2010) and GIS approaches (e.g., Hamzei et al., 2020) distinguish between geographical and anthropological types of facets. For this reason, we propose that the place concept “Sydney” includes the geographical facet ***extension’***, and the anthropological facet *citizens-**opinion’***. We can thus define a basic ontology of facets by introducing *G* and *A* as *sub-types* for geographical and anthropocentric facets, respectively. We assume that these sub-types form mutually exclusive sets (i.e., *G ⊓ A = ∅* holds), and that they jointly form the general type of facets *Φ* (i.e., *G ⊔ A* = *Φ* holds). We represent these types via sub-scripts: ***extension***_***g***_***’*** and ***citizens-opinion***_***a***_***’*** are typed versions of these facets. We can therefore enrich place concepts with an ontology of facets.

**Figure 9:**
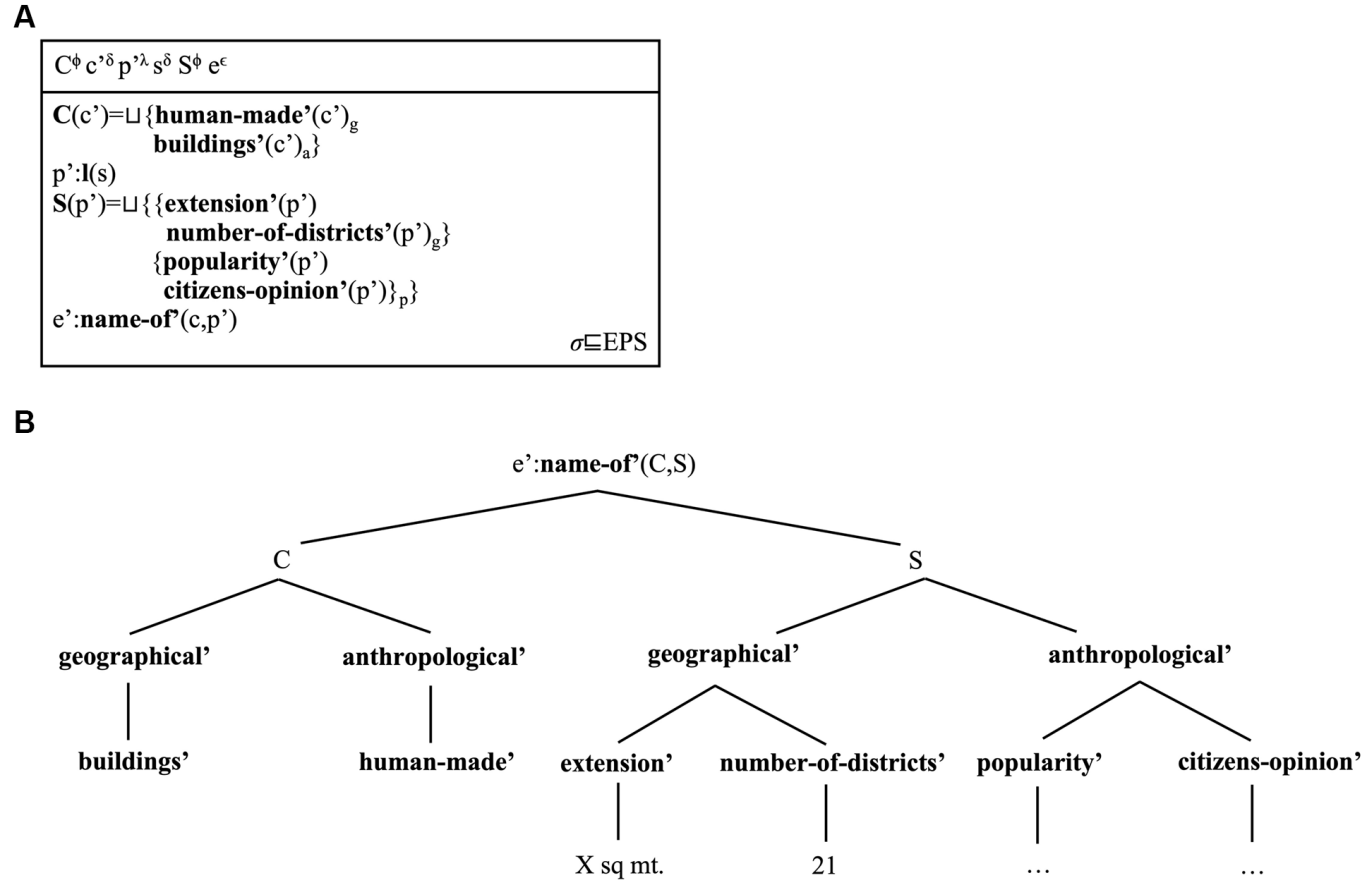
The place concept in **(A)** represents facets in a compressed form. The frame-theoretic representation of the facets in **(B)** decompresses their hierarchical structure. The place concept for Sydney includes two geographical and two anthropological facets. The first set in the DRS corresponds to the set union of the geographical facets; the second set, to the union of anthropological facets. Assigning specific values to these facets/attributes may add a further level. For instance, Sydney includes 21 districts, so one can connect this value to the corresponding frame. Connecting edges between nodes can be read in either direction. From bottom to top, they represent hierarchical relations between facets and their (super)-types. From top to bottom, they represent relations between attributes and their values. *Sydney* as a place name acts as a linguistic label for this whole frame; when we use this place name, we can refer to this complex conceptual frame.

**Figure 10:**
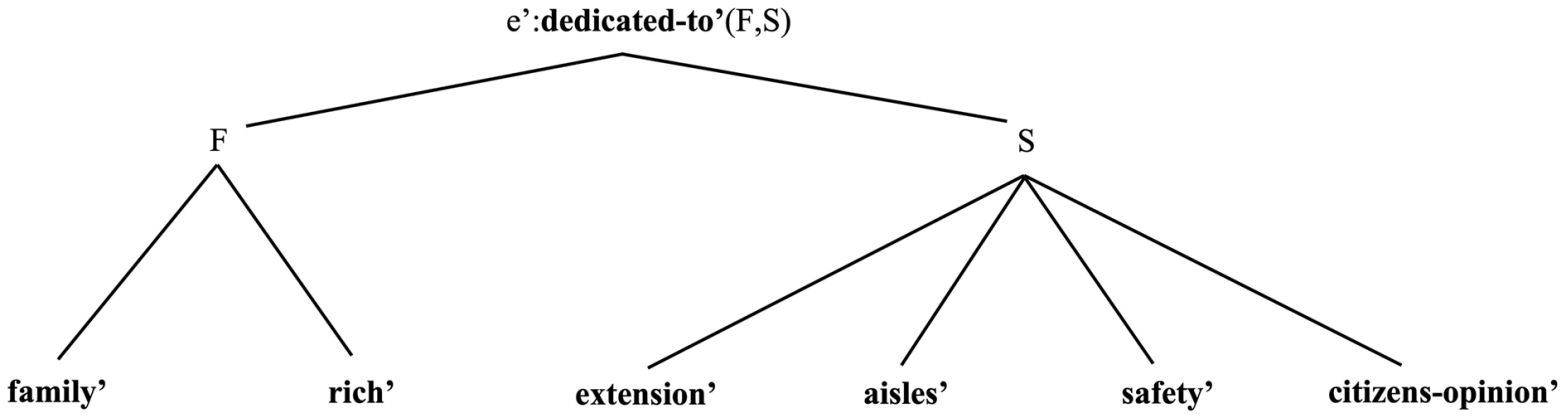
Frame-theoretic representation for the “Pitt Street” concept. Note that concepts can undergo a form of compositional facet unification (“feature unification” in frame theories: Löbner, 2014, 2021). The concept “Pitt” and the concept “Street” are represented via their structured facet referents, ***F*** and ***S***. These concepts enter a naming relation, as they individuate two different concept types (respectively, individual and Place types). The relation ***dedicated-to’*** represents this unification process, and also establishes that the compositional concept “Pitt Street” includes facets belonging to both concepts. The event *e’* represents the naming event that sanctioned this relation between Family *concept* and Place *concept*, establishing the etymology of this name.

**Figure 11:**
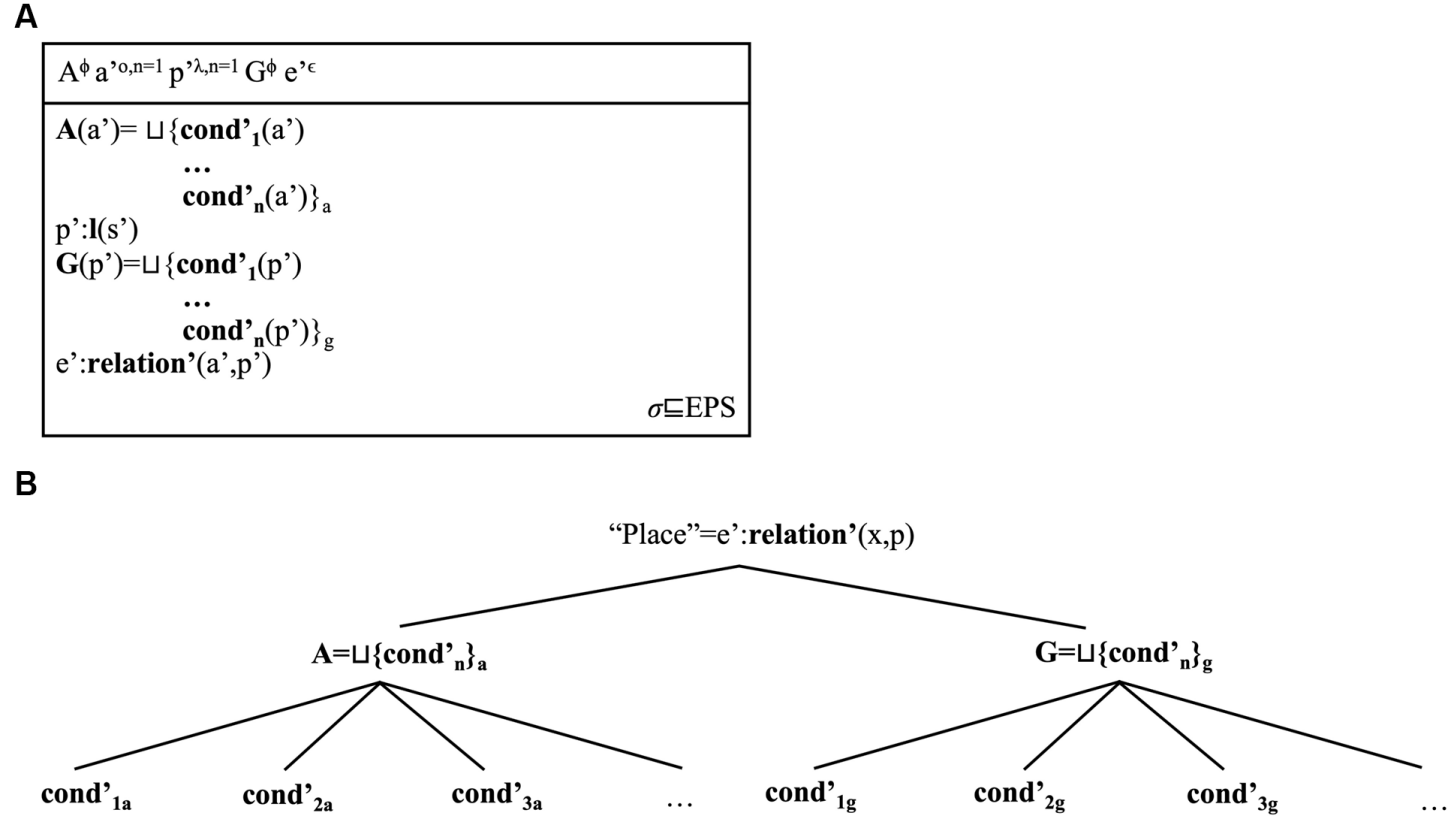
Singular place concept (s): general template in DRT format [i.e., **(A)**] and frame format [i.e., **(B)**]. As the template shows, geographic facets individuate the specific place referent *p’* that a concept describes, and anthropocentric facets individuate a possibly distinct second referent *a’*. In the case of “Pitt Street,” the second referent is the aforementioned family after which the street is dedicated. In the case of “Sydney,” it is the city as the complex human artifact (and object) that is located in this place. “Place” thus becomes the relational concept that binds objects and locations, but also the manifold facets individuating these mental referents, here represented as union sets of more basic facets/conditions. Via frames we can potentially model all the semantic (i.e., conceptual and linguistic) representations of place concepts to the effect of faithfully representing individuals’ meanings for places. Our figures simply offer an overview of the possible structures emerging from these processes.

We can now use this ontology to model relations among place names. Hence, we can use our linguistic model to capture relations among place concepts: define place networks as a reflection of the relations that individuals internalise about places. We show why this is the case via the conceptual DRSs for “Sydney” and “Pitt Street” in Figures 9, 10. Since we have specific templates for place concepts is that we can formulate a general template for place concepts, which we offer in Figure 11.

Figure 9 shows that *Sydney* and other bare place names can refer to complex Place concept frames and their hierarchies. Instead, Figure 10 shows that *Pitt Street* and other complex place names can include a certain division of labour. PlacePs seem to refer to geographical facets (e.g., a place belonging to the “street” type); NamePs, at least indirectly, to anthropological facets (e.g., the name being dedicated to a family). Once these words form a place name, their frames form the unified frame for the Place Name. Figure 11 therefore generalises this model to any place concept. It shows that place concepts involve different types of facets, which can then individuate the referents (objects, locations, individuals related to places, events) shaping these concepts. Hence, our model of place concepts can potentially capture any type of meaning (e.g., geographical, anthropological, historical) associated to places by re-interpreting DRSs as frames.

**Figure 12:**
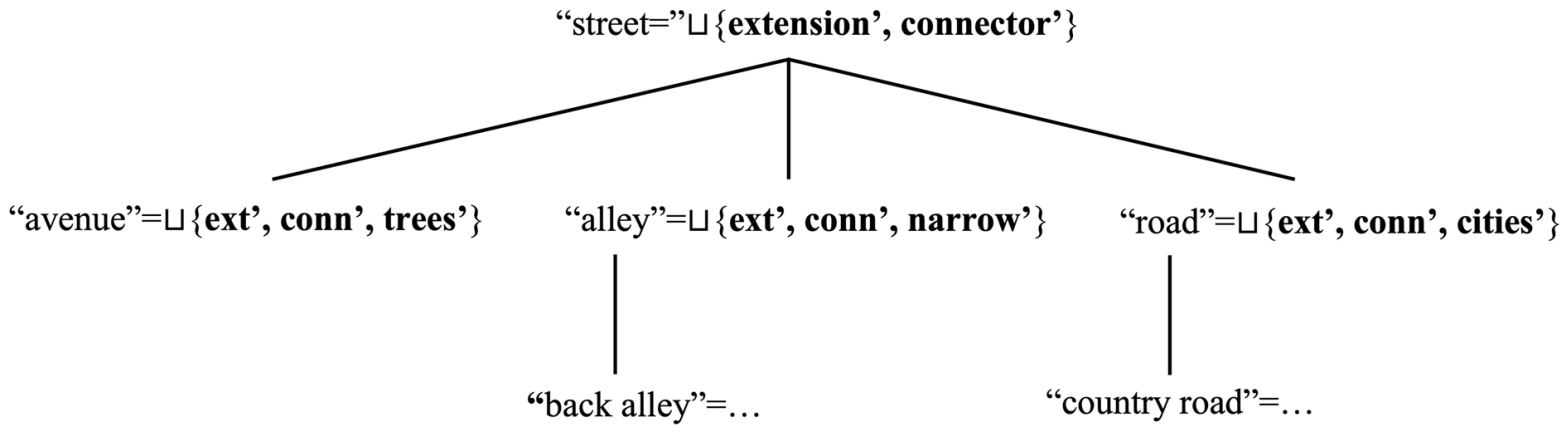
In this hierarchy, we represent concepts and the facets potentially distinguishing these concepts. We propose that “street” minimally includes the facets ***extension’*** and ***connector’***: a street is a place with an extension and connecting other places. We then propose that the concepts “avenue,” “alley” and “road” include at least one more facet distinguishing one concept from the other place concepts. For instance, avenues usually have trees, so the “avenue” concept includes the facet ***trees’***. Alleys are often narrow, so the concept “alley” includes the facet ***narrow’***. Roads can sometimes connect different cities, so the concept “road” includes the facet ***cities’***. These three concepts are thus sub-ordinate to the “street” concept, because their condition sets include more facets and therefore individuate smaller sub-sets of places (e.g., “avenue” ⊑ “street” holds because *⊔{**ext’**, **conn’**, **trees’**} *⊑ ⊔*{**extension’**, **connector’**}* also holds). The Supplementary file contains more details on Figures 9–12.

The model can also capture place networks as follows. In standard dictionary definitions, *avenue*, *alley* and *street* are terms referring to place types definable via various facets (e.g., spatial extension, ability to connect other places in cities; Ursini and Samo, 2022). However, avenues usually include the presence of trees, aisles and other spaces for the leisure of walkers; alleys are narrower, secondary streets. One can also consider back alleys and side alleys as possible sub-types of alleys. Roads may be streets that may stretch beyond cities, unlike streets and alleys. The terms *avenue, alley, road* and *street* can thus refer to concepts that seem related via the different facets shaping their structure. Such hierarchical relations can be modelled via the sub-type relation “⊑”: via facets, we model “avenue,” “road” and “alley” as conceptual sub-types of the “street” concept. Hence, the lexical hierarchical relations between *alley*, *avenue*, *road* and *street* reflect how individuals conceptualise different types of places and relate them via their increasingly descriptive content. We show the emerging set of hierarchical relations from this example in Figure 12.

**Figure 13:**
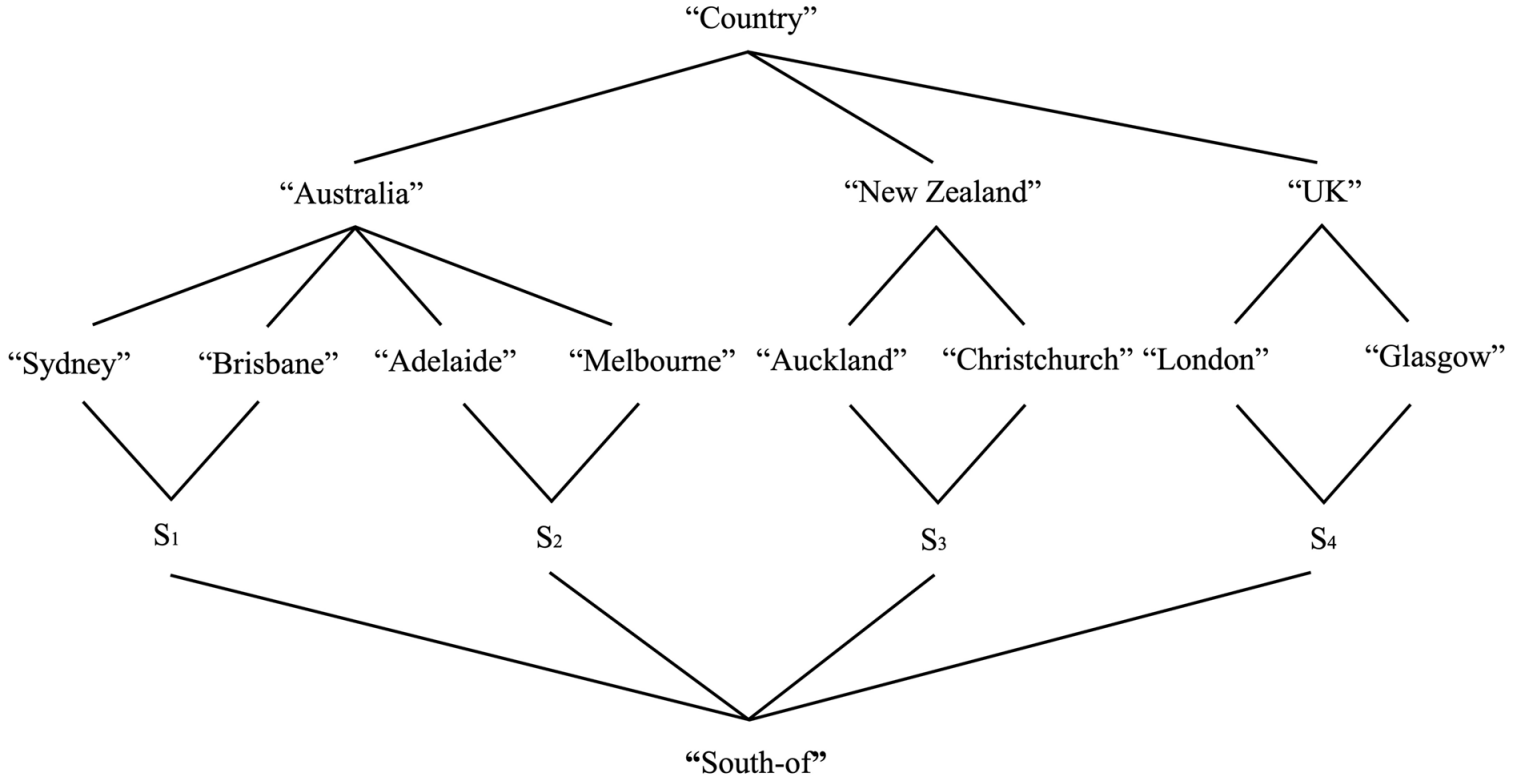
The model shows that “Sydney,” “Brisbane,” “Adelaide” and “Melbourne” are singular place concepts that are part of the “Australia” concept, and thus indirectly part of the “Country” concept. This concept is a sub-type of the “place” singular concept (not represented in the figure for space reasons). Other sub-types we introduce in our toy model are “New Zealand” and “UK,” as sub-types of the “Country” concept. The “South-of” spatial relation is a concept that can have multiple realisations or states (e.g., *s_1_* for the relation between “Sydney” and “Brisbane”). However, while “Sydney” and “Brisbane” are hierarchically linked to “Australia” (i.e., both singular place concepts are related to the “Australia” place concept), their spatial relation is mediated via a different concept and domain. For this purpose, we add nodes explicitly representing the states associated to each relation as a concept (cf. Landman, 1991: Ch. 6; Landman, 2000: Ch. 8; Ganter and Wille, 1998: Ch. 4; Krifka, 1998; for discussion on this technique).

**Figure 14:**
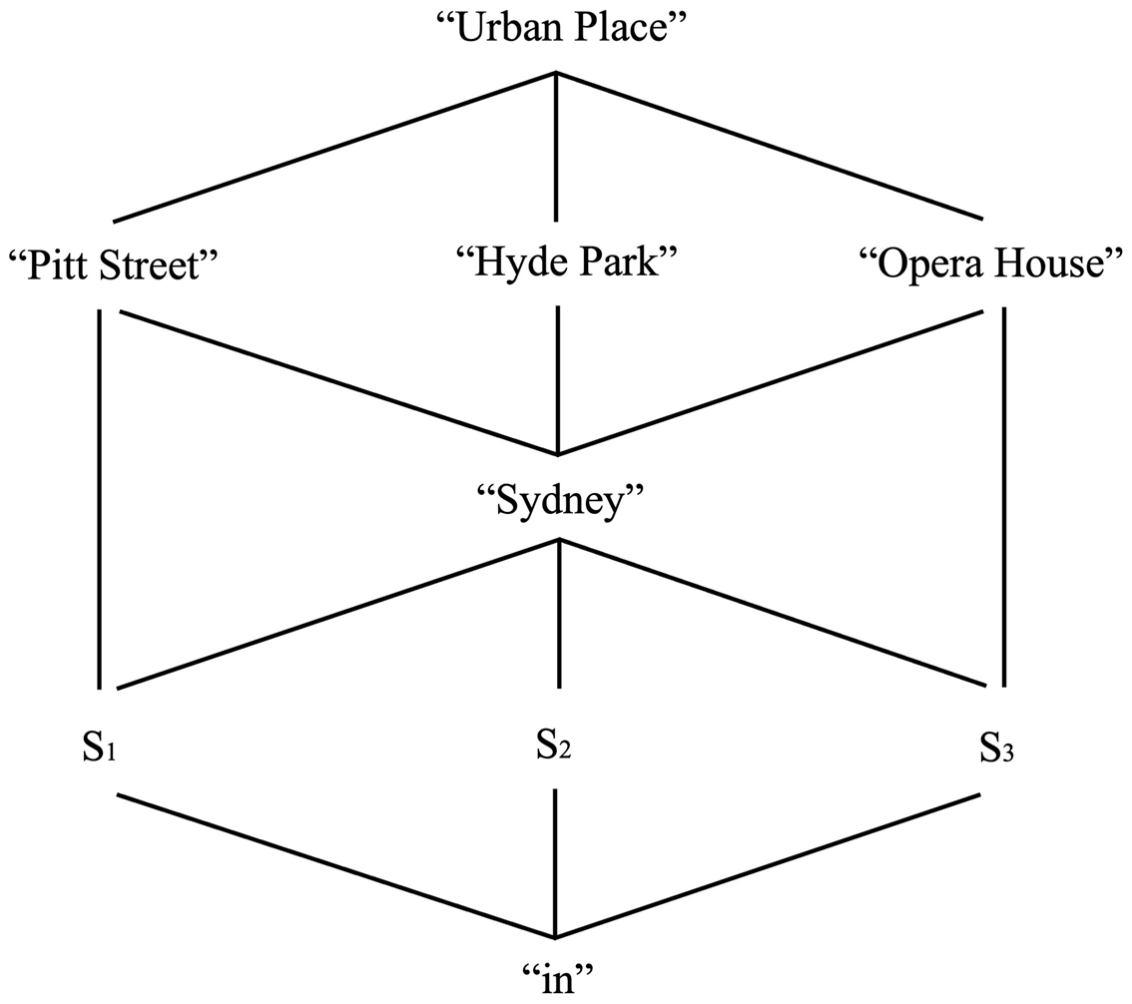
The hierarchy shows that “Pitt Street,” “Hyde Park” and “Opera House,” among other place concepts, can be defined as sub-types of the “Sydney” concept: in this case, parts of this city that are also distinct (sub-)types of urban place concepts. We can add the concepts explicitly representing these relations, to model the emergence of the “in” relational concept. Thus, *s_1_* represents in abbreviated form the state described by relation *s: **in’**(Pitt-Street, Sydney)*. This relation represents the fact that the place concept “Pitt Street” and the place concept “Sydney” are connected via the relational spatial concept “in,” and thus describe a spatial state *s_1_*. This relation, in turn, considerably abbreviates the relation that the sentence *Pitt Street is in Sydney* in (2) denotes. The set of states that one can define between “Sydney” and the other place concepts via this ‘inclusion’ relation forms part of the denotation of the “in” concept in our toy model: other “in” states can be defined accordingly, via relations between “Sydney” and other Places being “in” this city (e.g., “Hyde Park,” “Opera House”).

We now move to lexical relations among place names. Adpositions provide our next test (e.g., *in, South of*, *in front of*), because they can reflect the spatial, relational organisation of place concepts. For instance, English *in* introduces a relation in which one place’s location can be included in a second place’s location [e.g., Pitt Street being in Sydney, cf. (2)]. Instead, *South of* or *in front of* introduce relations between two place concepts via a third location and the reference system it introduces [e.g., Sydney being South of Brisbane, cf. (6)]. Consider thus Figures 13, 14.

As Figure 13 shows, we can represent “Sydney” and “Brisbane” as Place concepts that are part of a network including “Australia” and “Country” as super-types. At a coarse-grained level of conceptual resolution, we represent the fact that these two cities are located in Australia, a country and therefore a more abstract place concept. Once we individuate these concepts, we can connect them to the “South of” relational concept by individuating the state (i.e., eventuality) in which the first city is located to the South of the second city. We represent the different natures of this relation via a graphical choice. Hyponym relations can be read in a top-down fashion; spatial relations, in a bottom-up fashion. We operate a similar analysis in Figure 14, which shows how Place concepts can be related via the “in” concept. We can show that “Sydney” as a place concept can also enter in mereo-topological relations with other place concepts (e.g., “Opera House”), mirroring spatial relations among places.

Let us summarise our achievements so far. Our model of place names can capture their key grammatical and lexical properties. These properties reflect the ontological properties of the place concepts to which these names are connected. Place concepts can be organised into networks according to the category-internal relations among their facets. These relations may be realised as lexical relations among place terms (e.g., *street*, *avenue* and so on). These relations can also be realised as lexical relations (e.g., ‘South-of”) among place names, mediated via verbs and adpositions. We have therefore defined an ontology for place networks, as proposed in the psychology (e.g., Scannell and Gifford, 2010) and GIS literature (e.g., Purves et al., 2019).

#### 5.3.4. Multiple place representations and shared models: prolegomena to place in discourse

**Figure 15:**
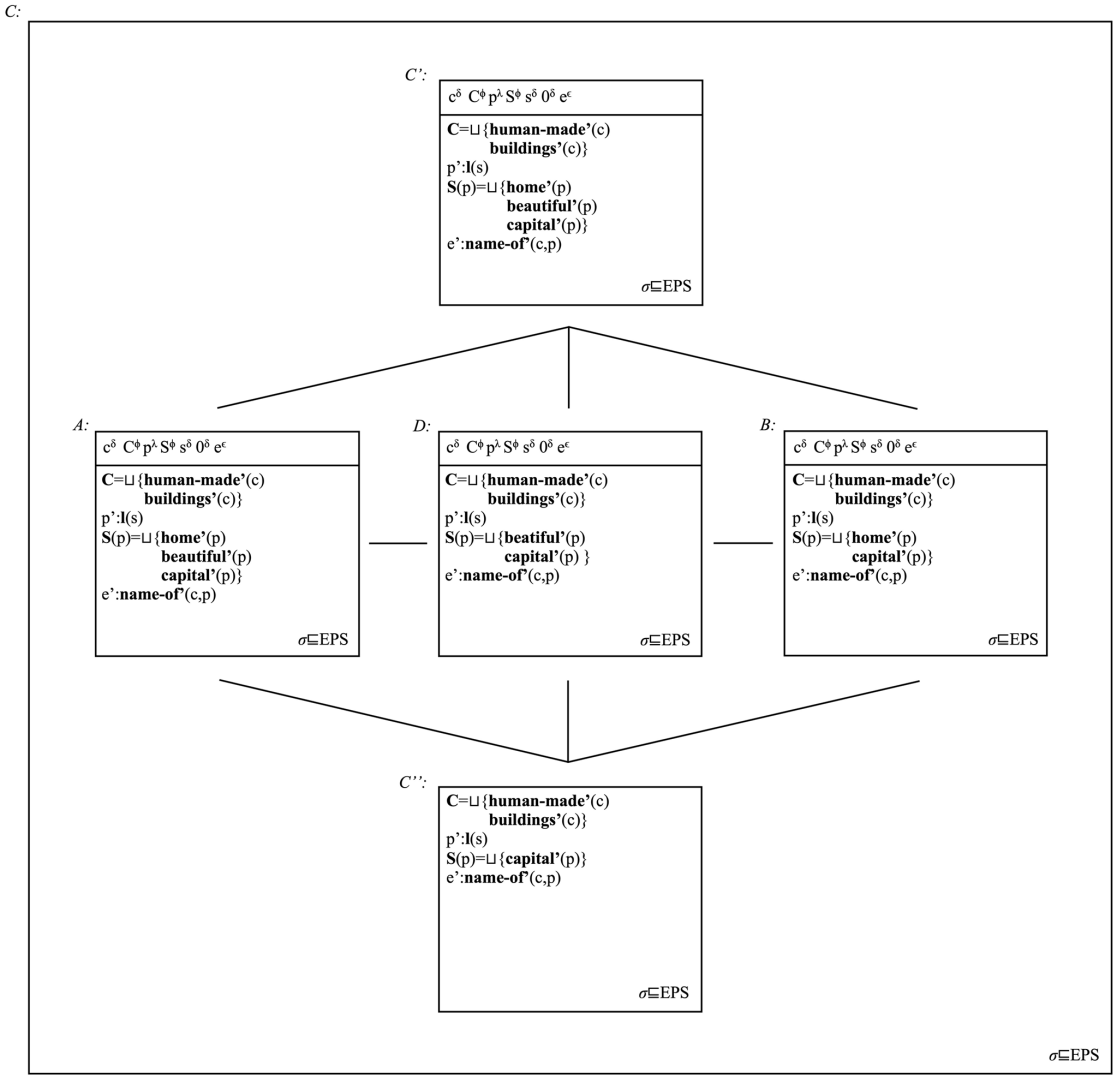
We use simplified DRSs for “Sydney,” to maintain our discussion compact (cf. Figure 5). The Supplementary file contains a more thorough discussion of the formal properties of this context. Horizontal edges are akin to “vicarious anchors” (Kamp, 2021, 2022) or “perspective relations” (Heller and Brown-Schmidt, 2023). In Figure 15, they represent the fact that individuals may connect their own DRSs for places to DRSs that other individuals may have about the same places under discussion. See the Supplementary file for more information.

In this section we show how our model can be used to represent inter-subjective place concepts in discourse. As discussed in Sections 2–3, individuals can have subtly different place concepts and forms of attachment representing one or more places. Such inter-subjective differences can emerge in how individuals discuss places in social media and other geo-tagged formats (e.g., Chen et al., 2018; Hamzei et al., 2020; Maren et al., 2020). In general, individuals can develop different though co-existing perspectives in discourse and compare these perspectives as a discourse unfolds (Heller and Brown-Schmidt, 2023; and references therein). In our specific case, individuals can discuss and present personal views/concepts of place and attachment relations. Individuals can simultaneously entertain inter-subjective concepts of these places, representing how other individuals conceive these places. One example involving “Sydney” as a place is as follows (cf. Figure 15 for the model).

Imagine three individuals that discuss their opinions of Sydney in an on-line conversation on TripAdvisor about “Sydney, capital of New South Wales.” As the conversation unfolds, the individuals *A*, *B*, *D* discover that they have subtly different views of this city. We offer an example of a conversation approximating such an exchange of opinions in (16).

(16) *A: I really find Sydney a beautiful*
      *city, one I can call home.*
  *B: Yes, Sydney is now also my home,*
      *though I was not born here.*
  *D: Beautiful, yes, but my home*
      *remains Auckland I guess.*

These opinions can share some factual knowledge, but each individual may have different feelings and thus forms of attachment towards this city. Individual *B* considers Sydney home (cf. the facet/condition ***home’***); individual *D* considers Sydney beautiful (cf. the facet ***beautiful’***) but not home; individual *A* considers Sydney home and a beautiful city. Given the title of the thread, all three individuals may also tacitly acknowledge that Sydney is the capital of the Australian state of New South Wales (i.e., the facet ***capital’***). All three individuals also indirectly agree that their respective “Sydney” concepts differ and are related to the city of Sydney, though some common aspects (i.e., facets) certainly can be defined.

We can model this shared opinion as the minimal discourse context *C’*. This context may represent a part of the on-line discussion, e.g., a post in which all three individuals acknowledge their common views on Sydney (i.e., the facets ***name-of’***, ***capital’***). Individuals can then consider each of their respective “Sydney” concepts as intermediate, possibly partial views on this city and the facets defining it. The horizontal edges connecting these individual relations (e.g., *A:DRS* to *D:DRS*) represent this form of inter-subjective understanding of the “Sydney” concept. A third emerging possibility is that individuals can jointly develop a “Sydney” concept encompassing all alternative views (i.e., *C’*). We can represent place concepts that individuals share as a group as the (set) union of their subjective views on a Place.

These logical possibilities correspond to different aspects of this mini-discourse and its context (cf. Heller and Brown-Schmidt, 2023, p. 4). In this context, each individual contributes distinct though partially overlapping views (i.e., concepts) of Sydney, and discusses how these views can differ from one another. They can agree that Sydney is the city they are talking about and, tacitly, the capital of New South Wales. They also display different forms of attachment to this city and concept, since they are willing to share and compare their views in this discussion.

Our model can now capture place attachment relations and their flexibility across individuals and groups (Scannell and Gifford, 2010). This result is achieved by showing that these relations are shaped by how individuals can define “senses of place” (i.e., conceptual content via facets) in their subjective representations of place concepts (i.e., our DRSs). Therefore, this analysis has three important consequences. First, it allows us to fully reconstruct place as a subjective, individual-oriented concept (e.g., Cresswell, 2014). Second, it permits us to show that inter-subjective place concepts can be defined in volunteered information contexts (e.g., Sui and Goodchild, 2011; Chen et al., 2018). Third, it also captures the fact that groups of individuals can share models that do not necessarily involve identical place concepts (Mohammed et al., 2010; Maren et al., 2020; Mocnik, 2022; Heller and Brown-Schmidt, 2023).

## 6. Discussion

We believe that our proposal offers key solutions to the recalcitrant problems outlined in the introduction. We expose these solutions via five discussion points.

First, we offer a novel morpho-syntactic account of place names. This account solves the problem of “category conflation” emerging in previous generative models (e.g., Svenonius, 2010). We model place names as corresponding to either phrasal compounds or bare NPs (cf. Köhnlein, 2015; Schlücker and Ackermann, 2017). This analysis hinges on identifying two morpho-syntactic categories: Place as a classifier category, Name as a content category. These categories form place names as an NP sub-type, distinct from other NPs (e.g., indefinite *a park*) and from PPs (e.g., *in a park*). We then embed our novel proposal in current formal (generative) treatments of proper names and NPs (e.g., Anderson, 2007; Acquaviva, 2019). As a welcome result, we can account increasingly complex places names (e.g., *New South Wales*, *Pitt Street* and *City of Sydney*), but also “bare” place names (e.g., *Sydney*). This is the case because we offer a generative, recursive and empirically justified morpho-syntactic analysis of place names.

Second, we offer a novel semantic account of place names. This account solves the problem of place names’ content being reduced to their referential properties (e.g., Coates, 2017). We use a multi-modal DRT analysis to achieve this result (cf. Pross, 2010). We assign a semantic interpretation to place names that captures their descriptive content *and* referential potential, via DRT’s treatment of proper names (Kamp, 2015; Maier, 2017). According to this interpretation, place names have senses that allow individuals to refer to places via the rich descriptive content they associate to these places. We also show that place names as NPs introduce reference to the baptism events in which a place receives its name (Kamp, 2022). We thus formally reconstruct accounts that address how individuals associate place names to rich senses (e.g., Blair and Tent, 2021; Perono Cacciafoco and Cavallaro, 2023). More importantly, we can capture the compositional contribution of the categories forming place names (e.g., *Pitt*, *Street* in *Pitt Street*). We also reconcile descriptive and referential theories of proper names under one multi-modal DRT model (cf. Kamp et al., 2011; Kamp, 2022).

Third, we develop a formal treatment of lexical relations among place names that reflects places’ conceptual relations (cf. Reszegi, 2020a,b). This account solves the problem of offering a full-fledged treatment of place networks as conceptual/semantic relations among place names and the place concepts they reflect (cf. Purves et al., 2019). We achieve this result by extending our approach with a frame semantics analysis of facets and place concepts (e.g., Löbner, 2014, 2021). We show that place names and terms (e.g., *alley* and *street*) can enter hierarchical types of relations in conceptual networks. We thus connect standard insights from lexical semantics (e.g., Cruse, 2000; Hanks, 2013) with novel results in GIS (e.g., Purves et al., 2019; Ursini and Samo, 2022). We also reconstruct a view of place names implemented in gazetteers as nodes of complex networks (e.g., Hill, 2006: Ch. 4; Hu, 2018). Overall, we sketch an initial formal model of the lexicon of place names (Reszegi, 2020a,b). Hence, we account why and how place names can refer to places, their facets and their relations.

Fourth, we solve the problem of having a full-fledged and cognitively plausible linguistic theory of place names. We model place concepts as domain-general DRSs: enriched versions of the geo-atoms found in GIS frameworks (e.g., Kuhn, 2012; Hu, 2018). We then offer a formal treatment of place concepts that is consistent with their analysis in geography and psychology (e.g., Cresswell, 2014; Manzo and Devine-Wright, 2021). We achieve this result by modelling facets, the building blocks of place concepts (e.g., Canter, 2012; Hamzei et al., 2020), as conditions forming conceptual DRSs. We then connect facets to a rich ontology underpinning psychological models (e.g., Scannell and Gifford, 2014; Seamon, 2018) and GIS formal models (e.g., Purves et al., 2019; Hamzei et al., 2020). We thus reconcile linguistic analyses of place names with non-linguistic analyses of place as a general concept with manifold declinations (e.g., “city,” “street,” ‘Sydney” and so on).

Fifth, our model achieves a considerable degree of inter-operativity. It connects linguistics, psychology and GIS via the notion of place, thus reaching a theoretical *desideratum* (e.g., Ballatore, 2016). This is a consequence of DRT integrating apparently opposite (e.g., cognitivist and formalist) perspectives (cf. Hamm et al., 2006; Pross, 2010; Maier, 2017). The model can also achieve a degree of inter-operativity with cognitivist models of communication (e.g., Heller and Brown-Schmidt, 2023; cf. also Maren et al., 2020; Mocnik, 2022). This is the case because our model also uses reference relations and multi-modal representations as the central tools to represent cognitive con tent. Considerations are however offered in the Supplementary file, due to length requirements.

## 7. Conclusion

The goal of this paper has been to propose a linguistic model of place names (e.g., *Sydney*) integrated with a conceptual model of place (“Sydney”). We have shown that such an account is possible once we offer a formal account of place names’ morpho-syntactic and semantic properties. Our account models place names as a sub-type of NPs involving rich descriptive content that can refer to the subjective or inter-subjective place concepts that individuals entertain in discourse. Therefore, our account can solve the recalcitrant problems illustrated via (1)–(3), (6)–(8), and (16). We obtain this result by introducing DRT as a multi-modal theory of mental content and as a variant of first order logic that is compatible with other frameworks. We then build a full-fledged linguistic and psychological model of place names and place concepts on this framework. Overall, we suggest that formal linguistics can offer key inter-disciplinary insights and tools to platial research (cf. again Tenbrink, 2020). This can be the case, however, insofar as linguistic analyses of place names maintain inter-operativity as a clear-cut research goal.

The account also invites the observation that other problems now become tractable (e.g., formal, cross-linguistic analyses of place names, cf. Köhnlein, 2015), though still outstanding. The approach also obtains at least two interesting secondary results. First, we may have the prolegomena of a novel model of adpositions and their senses, fully compatible with our model of place names (cf. also Ursini, 2020; Ursini and Tse, 2021). Second, the model also appears compatible with recent approaches to place names based on construction grammar (e.g., Leino, 2007, 2011) and cognitive linguistics (e.g., Sjöblom, 2011; Reszegi, 2022). Though these frameworks use different assumptions from the generative and DRT assumptions we implemented in this paper, they also propose rich representational analyses of place names. Third, our model may be consistent with multi-modal models of memory (e.g., Tulving, 2002; Cowan, 2005; Bush et al., 2014). In such models, memorising information about events and individuals occurs by tracing their “place” in the world, via dedicated sets of so-called “place cells.”

Our model thus seems potentially extendable to domains and frameworks that may offer further views on place names and places. However, we leave the exploration of these extensions for further research.

## Data availability statement

The raw data supporting the conclusions of this article will be made available by the authors, without undue reservation.

## Author contributions

F-AU collected the literature (psychology, linguistics, GIS), developed the DRT and frames parts of the article, and wrote the article. YZ collected the literature (psychology, linguistics), developed the frames parts of the article (visual notation, diagrams, figures), and edited the article. All authors contributed to the article and approved the submitted version.

## Conflict of interest

The authors declare that the research was conducted in the absence of any commercial or financial relationships that could be construed as a potential conflict of interest.

## Publisher’s note

All claims expressed in this article are solely those of the authors and do not necessarily represent those of their affiliated organizations, or those of the publisher, the editors and the reviewers. Any product that may be evaluated in this article, or claim that may be made by its manufacturer, is not guaranteed or endorsed by the publisher.

## Footnotes

^1^We omit representing temporal information, e.g., the sense associated to the simple past *landed* in (11).

^2^We gloss over place names including morphologically complex forms (e.g., *Man-chester*); length requirements prevent us from addressing these classes. See however Köhnlein (2015); Ursini (2016); Perono Cacciafoco and Cavallaro (2023: Ch. 3) for discussion.
