# Supplementary material for: Place and place names: a unified model
Source: Front Psychol. 2023 Sep 8;14:1237422. doi: 10.3389/fpsyg.2023.1237422 (PMC10539907; doi:10.3389/fpsyg.2023.1237422)
Supplement: Supplementary file 1 [file Data_Sheet_1.pdf]

Supplementary notes to Sections 5–6

In this supplementary file we offer some further discussion about the formal aspects of our proposal, while also offering additional references and connections to relevant previous works. Readers who are not interested in these aspects may safely skip this file; the main text contains all the relevant aspects of the proposal.

Figure 2: Anchors and their properties

Anchors, as defined in the main text, connect referents and conditions also in a recursive manner (e.g. we have $f(ref^{\tau})ref^{t}$, $f(Cond^{\tau})=Cond^{t}$, and so on). This is possible only when one DRS is accessible to another DRS. *Accessibility* is thus defined as a relation that in turn is defined as a pre-order, i.e. a transitive and reflexive relation between DRSs. If we have a sequence $S=<K,K',K'',\ldots>$ of DRSs, then each DRS is accessible to itself and the following DRSs. The *accessible domain* of a DRS *K* is the universe of discourse of each DRS accessible to *K*. In the sequence *S*, *K''* can access the referents in *K'* and *K*, aside its own referents; the DRS *K'* the ones in *K* and its own; *K*, only its referents. More complex accessibility conditions can be defined via the syntax of DRT (cf. Kamp et al. 2011: Ch. 0; Geurts, Beaver & Maier 2020), though we do not need to explore this matter in this paper.

Anchors can bind referents across DRSs provided a suitable mapping between different types is defined. Fig. 2 in the main text, for instance, includes a perceptual DRS 1, a Place DRS σ and a linguistic DRS *s* that form the sequence $S_1=<1,\sigma,s>$. In our case, *s* can access the referents in σ and *1*: a linguistic referent can access conceptual (place) and perceptual (visual) referents as its sources. Note, once more, that conditions also become bound across DRSs, and thus are subject to the accessibility constraint. As we have discussed in the main text, this leads to the formation of anchors connecting concepts and linguistic referents (e.g. $ANCHOR<\sqcup\mathbf{Cond}_n,P>$). This is possible because linguistic referents can access conceptual referents via opportune mappings. This is possible also because our Boolean approach to referents allows us to define unions and intersections of referents (i.e. we can define $\sqcup\mathbf{Cond}_n$ as a possible entity in our ontology).

Anchors are mappings across domains, so they should preserve restrictions over these domains (e.g. conditions individuating referents: cf. Landman 1991: Ch. 2; Kamp et al. 2011: Ch. 6; Ursini 2011). By definition, this is possible only if the restrictions become part of anchors. Given the identity between this structured referent and a (union) set of facets, the anchor $ANCHOR<\mathbf{S}^{\pi}=\sqcup\{\mathbf{cond}_n\}(p'),\mathbf{name'}(p)>$ holds. Thus, the sense of a place name acts as a label for the manifold of facets that the corresponding place concept describes. To see why this is the case, we further clarify the anchors in Fig. 2.

The external anchor $ANCHOR<\alpha,x'>$ connects a source perceptual referent for the plane with the floater object referent *x'*, presenting this object at a conceptual level. The internal anchor $ANCHOR<x',x>$ connects conceptual referent with discourse referent. From this anchor, $ANCHOR<\mathbf{P}(x'),\mathbf{plane'}(x)>$ is derived: the sense for the NP *(the) plane* label the set of facets identifying a "plane" concept. Place concept referents are instead connected as follows. The internal anchor $ANCHOR<p',p>$ connects a source conceptual referent (i.e. *p'*) with a floater discourse referent (i.e. *p*). The external anchor $ANCHOR<\beta,s>$ connects a conceptual referent, and by transitivity a discourse referent, with a perceptual referent. Thus, a picture of Sydney as a place activates a flow of perceptual information for the city of Sydney as an object. It is at the conceptual, EPS layer that place concepts referents enter the frame: a location function $\boldsymbol{l}$ assigns a spatio-temporal location to Sydney as an object occupying this location.

The anchors between structured referents hold for the following reason. If *ANCHOR<p',p>* holds, then any conditions individuating *p* and *p'* must preserve this relation (i.e. we have *ANCHOR<**f'**(p'),**g'**(p)>*). We then have *ANCHOR<e',e>*: this is an anchor between a conceptual representation of a landing event (represented via the referent *e'*) and its linguistic realisation, described via the event *e*. The corresponding anchor *ANCHOR<**land**(x',y'),**land'**(x,y)>* connects the verb *land(ed)* to the relational concept it describes. As we offer this concept in compressed form, only the referents differentiate the two conditions. It also connects the event concept it describes (call it "land(ing)") to the language-specific form (the verb "land(ed)") describing this event (type) concept. As we explain in the paper, our notion of reference is a mediated one: reference to mind-external entities is always mediated via mind-internal entities. This view is consistent with the view offered in Mental State DRT (Kamp 2021). Our use of anchors however differs from this framework in following a less detailed formalisation of anchors, and having anchor sets below rather than to the right of (content) DRSs.

It is also worth noting that the ontologies introduced in Section 5.2 are defined via sets of different referent types (or models) and *n*-ary functions that we can define over these referents (i.e. conditions). Conditions and referents can potentially be combined into more complex entities in discourse (e.g. structured referents, DRSs), via the two Boolean operations "⊔" and "⊓". We can then have "⊑", a general relation that can be restricted to referents, conditions and DRSs (e.g. "type-of" for facets). An ontology *Σ=<Ω,Λ,E,Φ,⊔,⊓,⊑>* is thus an ontology of non-linguistic, spatial entities from which we can recursively define structures forming various types of concepts. We focus on singular place concepts in our paper, but we also show how to define (other) spatial concepts such as "front" (cf. Section 5.3.2 and this file). An ontology *S=<D,L,EV,P,⊔,⊓,⊑>*, a linguistic ontology from which we can recursively define linguistic senses assigned to place names. As in Frame-based theories (e.g. Löbner 2014, 2021), one can ultimately build (or: recursively enumerate) concepts and linguistic entities by assuming that DRSs are attributes, and conditions and referents are values. This point is further expanded in Section 5.3.3, and in the notes to Figures 9–14.

Figure 3: Recursion in Place Names

The structures in Fig. 3 assume a hierarchical view of constituent structure: heads form a preliminary constituent with their complement phrase. For instance, in the structure in b. the silent head Ø projects an N category, combines or *merges*, in generative parlance, with the NameP *Shasta*, and forms the intermediate N' (N bar) *Ø Shasta* (cf. Cornilescu 2007; Acquaviva 2019). This constituent merges with a specifier phrase: the PlaceP *Mount*. The place name (i.e. a compound NP) *Mount Shasta* is thus formed. A place name like *New South Wales* includes recursion of PlacePs (i.e. *New* and *South*), plus recursion of nested NPs introducing these PlacePs. More complex recursive structures could in theory occur; nevertheless, empirical evidence for further degrees of recursion would become necessary.

Figures 4–5: Anchors and Place Names, Syntactic Considerations

The anchors below the discourse DRS *a* in Fig.4 read as follows. We omit referents in anchors and thus show these relations as holding over the conditions defined over the corresponding referents. The first four anchors (i.e. *ANCHOR<f,x>*, *ANCHOR<s',s>*, *ANCHOR<p',p>*, *ANCHOR<e',e>*) anchor referents across the two layers. The structural anchors (*ANCHOR<**ST**, **street'**>*, *ANCHOR<**PT**, **Pitt'**>*) emerge as *Pitt* and *Street* are terms labelling the two concepts forming the

concept associated to *Pitt Street*: a street named after some wealthy family from Sydney. *ANCHOR<**dedicated-to',R'**>* identifies this relation as the naming relation holding between the two aforementioned concepts. It represents the fact that though no overt word captures this relation, the place name *Pitt Street* as a single phrase refers to this naming relation and the event establishing it.

As the representations show, the labelling function of place names' senses has a dual role (cf. again Kamp 2015; and references therein). When a place name lacks an overt (i.e. phonologically realised) exponent, we can assume that a PlaceP can introduce a referent and an identity condition ***Id'*** over this referent (cf. Kracht 2002, 2004). Thus, *Sydney* as a "PlaceNameP" introduces a relation between this place concept and some other referent in discourse related to this place concept, as the anchors *ANCHOR<**S,Sydney'**>* and *ANCHOR<**name-of',R'**>* show. In other words, *Sydney* and other place names lacking place classifiers do refer to place concepts, but do not state which type of place concept is at stake.

This analysis is consistent with the fact that bare place names may create ambiguities in retrieval tasks, as discussed in Section 3. If individuals use *Sydney* as a name for e.g. the city's Aussie rules team, retrieval software may erroneously interpret the name as referring to the city (e.g. Buscaldi 2011; Zhou et al. 2016; Chen et al. 2018). The place name *Sydney* introduces the "Sydney" concept in discourse and some other referent related to this concept, which may be the city itself. However, if other concepts are under discussion, only textual and/or extra-linguistic cues will clarify this matter. We thus predict that reference in place names is always layered and potentially ambiguous (again, Kijania-Placek 2021).

Figures 6–8: Anchors and adpositions, Syntactic Considerations

For Fig. 6, the anchors between spatial concepts and the prepositions (and parts thereof) expressing these concepts work as follows. First, *ANCHOR<**South',S**>* establishes the relation between the NP and a cardinal point as a "part" of Earth, defined via a reference system based on polar coordinates. Second, *ANCHOR<**l',loc'**>* establishes the relation between the LocP including this NP, and establishes reference to the corresponding "South" (location) concept. Third, *ANCHOR<**B,Brisbane'**>* establishes that *Brisbane* names a place with a certain set of distinguishing facets; *ANCHOR<**name-of',R'**>* is the fourth anchor, establishing that the naming event associated to *Brisbane* has a linguistic realisation. Fifth, *ANCHOR<**direction-of',R'**>* establishes that the PP includes a "direction" relation defined via a Place and a location introducing the relevant direction. Overall, the PP *South of Bribsbane* refers to a state, i.e. a type of eventuality in which this complex relation between a "South" location/direction and "Brisbane" as a place holds (cf. Haselbach 2017: Ch. 6). These locations also indirectly introduce reference to coordinate systems as complex relational concepts, and can then enter spatial relations computed with respect to other locations, including Places.

For Fig. 7, *ANCHOR<**front',F**>* refers to the concept individuating a "front" object. The (composite) *ANCHOR<**loc',l'**>* refers to the concept individuating the (intrinsic) location that a front occupies. Here we analyse *in* as a projection of the Loc head that overtly realises the ***loc'*** relation: this is a reflex of this preposition's ample polysemy and distributional flexibility (cf. Evans 2015). The ground NP *the table* thus refers to the concept defining the landmark object (i.e. we have *ANCHOR<**T, table'**>*). The anchor *ANCHOR<**location-of',R'**>* establishes that the whole PP refers to a state holding between a table and its front location, and thus it involves a "location-of" (conceptual) relation between two concepts.

Some further morpho-syntactic observations are as follows. As also mentioned in Section 3, though English lacks case morphology, in languages such as Finnish locative case markers can introduce the ***loc'*** function explicitly (cf. again Haspelmath 2019). Furthermore, the analysis of the relational head *of* sheds light on the subtle differences between place names as NP sub-types and other NP and PP types. In place names, this head projects the N category: it takes two nominal arguments (PlaceP, NameP), and refers to a naming event once an NP is formed. In prepositions, it projects the P category. It takes a prepositional and a nominal argument (i.e. LocP and NP), and refers to a spatial relation and its state eventuality once a PP is formed. Therefore, PPs and place names capture different types of relations and eventuality sub-types, though they both may include *of* or other relational adpositions realise these relations (den Dikken 2006; Köhnlein 2015). Adpositions and place names jointly form a joint "language of space and place" (cf. Levinson & Wilkins 2006: Ch. 1; Rybka 2015: Ch. 7; Tenbrink 2020).

Figures 9-12: Frames, facets and Place concepts networks

Frames can be conceived as graphs in which nodes represent cognitive units (e.g. concepts, words) and edges represent relations of some type (e.g. accessibility, hierarchy/sub-type, hyponymy). Frames have been extensively used in Head Phrase Structure Grammar to analyse linguistic signs (Pollard & Sag 1994; Sag, Wasow & Bender 2003; Sag et al. 2012; cf. also Löbner 2011, 2014, 2021). Our extension to DRT and generative syntax seems a theoretical novelty, justified by the need to model facets/conditions as recursively defined structures involving conceptual/semantic attributes and values (cf. Gieryn 2000; Canter 2012). Note that we can interpret DRSs as involving (set) unions or intersections of conditions. An interpretation based on set union hinges on starting from the basic conditions in a frame and then combining them to form a concept (i.e. we define relations from bottom to top). An interpretation based on a set intersection perspective hinges on starting from the complex, relational concept and then moving to the single conditions (i.e. we define relations from top to bottom).

Some clarifications about the notation for Fig. 9–10 are as follows. First, "Sydney" acts as a theoretical label and shortcut for this frame. One can use the pre-theoretical identity relation "Sydney"=*e':**name-of'**(C,S)* to represent that this and other singular Place concepts can involve rich conceptual relations and frames. Second, we omit referents in the frame representation for reasons of readability: the conditions ***buildings'*** and ***human-made'***, for instance, should read as ***buildings'****(c')* and ***human-made****(p')*, respectively. Third, we partially abuse notation by representing the naming event and its corresponding relation as *e':**name-of'**(C,S)*: a relation between feature sets/frames rather than their corresponding referents. We thus represent the fact that "Sydney" is a name assigned to an object identified via its facets (i.e. complex, human-made), the facets of the unique place it occupies (e.g. extension), and the event in which this relation was officially sanctioned. Therefore, frame-theoretic accounts and DRT converge in their analysis of Names as (structural) labels (cf. Kamp 2015, 2022; Löbner 2021).

The type of lexical/conceptual relations outlined in Fig. 11–12 are in line with standard assumptions in lexical semantics (e.g. Cruse 2000; Hanks 2000, 2013; Kearns 2006; Murphy 2010; Riemer 2010). Specifically, they represent the standard assumptions that lexical items can realise conceptual relations in a language; conversely, conceptual relations determine lexical, hierarchical relations among lexical items. For instance, the hyponym relations *avenue*⊑*street*, *alley*⊑*street*, *road*⊑*street* hold: *avenue* is a hyponym of *street*, *alley* is also a hyponym, and so is *road*.

Therefore, the analysis of place names can shed light on both conceptual relations among place concepts and on lexical relations holding among place names (Ursini & Samo 2022). Note also that generic terms in the same "layer" of a hierarchy form near-synonym sets ("synsets"), i.e. terms of words differing in some minimal aspect of their underlying concepts (e.g. *alley*, *road*: cf. Fillmore 1982; Fillmore & Baker 2010).

A final observation is that these hierarchies/networks double as ontologies for place concepts and their types. One could use several frameworks to further develop such ontologies (e.g. Ganter & Wille 1998; Pustejovsky 1995; Asher 2011; Guizzardi 2005; Busse 2012; Cooper 2017; Löbner 2021). One could also assume that facets find linguistic counterparts in the type *pr* of properties, which can organise conditions as appearing in IRS (i.e. discourse) DRSs. Nuanced theories of properties and kind as their *denotata* certainly exist (e.g. Chierchia & Turner 1988; Chierchia 1998, 2010). Our conceptual and discourse ontologies seem to achieve a degree of symmetry. However, we leave a fuller exploration for the future.

Figures 13–14: Networks and their properties

In Fig. 13–14 we use *cross-dependency* hierarchies, i.e. hierarchies that organise the lexicon across different dimensions. Cross-dependency is achieved by having nodes (e.g. concepts, lexical items) potentially connected via several edges to form multiple relations (e.g. hierarchies). In Head Phrase Structure Grammar (e.g. Sag et al. 2012) and frame semantics (e.g. Fillmore & Baker 2010; Löbner 2014, 2021), these dependencies are often used to illustrate lexical and grammatical relations among different categories and constructs. In our specific case, we illustrate that place concepts can enter certain hierarchical relations realised as relations among their respective place names (e.g. "Sydney" as the place concept part of the "Australia" concept). The result is a minimal hierarchy of place concepts, represented via the nodes "Sydney" and "Australia" connected via a set of edges standing for conceptual relations. At the same time, place concepts can enter spatial relations (e.g. the "South of" relation), so the node "Sydney" is also connected to the node "Brisbane" via a different set of edges. Thus, the "Sydney" concept can enter in cross-dependency hierarchies, as it belongs to different hierarchies at once.

One could therefore define increasingly complex place *and* space networks in which concepts can be organised across different dimensions, in a manner similar to WordNet semantic networks (cf. Fellbaum 1998; Busse 2012). We leave this goal for future endeavours.

Figure 15: Inter-subjective contexts

The frame-theoretical representation shows that a minimal context *C'* can be defined via the set intersection of the three concepts. In set-theoretic terms, this corresponds to $C'{:}\sqcap Facet_{\phi}=\{\textbf{capital'}\}$. The maximal context *C''* can be defined via the set union of these concepts. In set-theoretic terms, this corresponds to $C''{:}\sqcup Facet_{\phi}=\{\textbf{capital}, \textbf{home'}, \textbf{beautiful}\}$. Individual disagreements thus correspond to the possibility that individuals do not accept more than one place concept (e.g. their own) to represent a place (cf. also Heller & Brown-Schmidt 2023; for a similar point). Let us note here that a possibility is that individuals only agree on one fact. That is, "Sydney" is a concept individuating a certain referent (i.e. that only the relation ***name-of'*** and the naming event it describes). We would reconstruct the core assumption of referential theories of names as a minimal case (e.g. Coates 2017). A place name like *Sydney* would only refer to an entity in discourse, but would otherwise not carry any content.

Concepts are reciprocally accessible by virtue of sharing facets and referents; hence,

they can form a complex context. The definition of formal contexts and accessibility relations as hierarchical relations among contexts can be developed via the tools of "Formal Concept Analysis" (e.g. Ganter & Wille 1998). In our case, the overall context can be approximately defined as $C=\sqcup\{C', A, D, B, C''\}$, i.e. as the sum of all possible shared concepts and resulting (sub-)contexts. That is, the context includes all possible concepts of "Sydney" that individuals can entertain. The fact that each individual's place concept is part of this context can be represented via the sub-type relation. Thus, $A\sqsubseteq C$ can be interpreted as individual *A*'s "Sydney" concept being a possible variant (type) of an inter-subjective, but singular "Sydney" concept, defined via the sum of all subjective facets ascribed to "Sydney" by each individual.

Note that in our model, collective attachment relations to place concepts need not to be "homogeneous". Formally, *A:DRS* and *D:DRS'* represent relations in which individuals have slightly different opinions of Sydney. The relation *C:DRS''* (i.e. a sum of Place concepts for multiple individuals in a context) can thus be a "composite" representation of this complexity. Nevertheless, homogeneous representations are certainly possible (cf. Scannell & Gifford 2010; Seamon 2018).

A consequence of this result is that online gazetteers and other place-based information can be treated as forming interconnected, complex contexts of interpretation for all of the place concepts that they present information about (e.g. Hill 2006; Mocnick 2022). If an individual reads different descriptions of the same place (e.g. the "Sydney" concept on FourSquare), the individual gains access to multiple but related versions of the same place concept. Individuals can find a shared sense for names to acknowledge that place names' senses reside in a community's use of the names. They are, to an extent, "distributed" senses (Kamp 2015: 298–308, 2021, 2022). In Mental State DRT, individuals are assumed to have access to these distributed senses via "vicarious anchors". Simplifying matters somewhat, $ANCHOR<A:DRS(\alpha),B:DRS'(\beta)>$ is defined if and only if $\alpha=\beta$. That is, vicarious anchors connect differing mind-internal DRSs by virtue of their reference to the same mind-external entities. Causal chains for acts of baptism can then be modelled via "historical" vicarious anchors: see, however, Kamp (2022) for details.

Finally, the "collective" sense defined via the possible, alternative senses can be conceived as a dictionary-like lexical entry for a place name. Words can have multiple, co-existing senses that correspond to the alternative understandings and uses of these senses amongst speakers (e.g. Hanks 2013, 2000). Our model seems to connect all these insights about place names as full-fledged vocabulary terms of a language in a unified, if distributed view.

Discussion: Further forms of inter-operativity

The model of place names outlined in the main text obtains three theoretical results that do not play a fundamental role in the paper, but nevertheless underline the theory's potential. First, the proposal extends DRT's approach to proper names in two aspects (cf. Kamp 2021, 2022). First, we treat place names as labels for "Entity Representations", rich and possibly multi-modal representations of "objects" (here, places). Second, we treat place names as part of conceptual and lexical networks in which individuals share and exchange naming conventions about entities in the world. The model therefore firmly makes this variant of Grounded DRT inter-operative with other DRT variants. We obtain two other forms of inter-operativity with frameworks beyond DRT, aside from those discussed in the main text. These forms can be defined as follows.

First, our model is consistent with representational, mind-internal theories of reference found in vision sciences and other cognitive disciplines (e.g. Multiple Object

Tracking: Pylyshyn 2003; Fodor & Pylyshyn 2014). In these models and our model, reference is always mediated via conceptual representations. Philosophical views, however, conceive reference as a direct mind-external relation between linguistic expressions (e.g. proper names) and “things in the world”, via baptism/naming social conventions (Coates 2017; Cumming 2019). Our model shows that complex place names (e.g. *Pitt Street*) refer to place concepts, and thus only indirectly to the places they represent. Furthermore, place concepts can include “nested” reference relations (e.g. to streets, families, naming events), and can vary considerably among individuals (Williams, Kuhn & Paihno 2012; Derungs & Purves 2014).[1] Our model thus shows that place names inform us about how reference in discourse emerges as a way for speakers to express their cognitive views about places, and thus share information about place concepts (cf. Tenbrink 2015, 2020a).

Second, our model is compatible with theories of word senses as “access points” to conceptual content (Evans 2015; Pietroski 2018). However, our model differs on two aspects. First, it suggests that word senses can reflect conceptual structure: generic terms (e.g. *street*) can explicitly realise the content of Place concepts’ facets (e.g. “Pitt Street”). Second, it suggests that concepts involve DRSs as multi-modal representations, of which linguistic DRSs represent the lexical, linguistic modality. Our model thus partially resembles the analysis of (lexical) concepts in Acquaviva (2008, 2022), restricted however to place names. Our model is also similar to the “mental files” model (Recanati 2012, 2016, 2021; cf. also Recanati 1993; Kamp 2021). In fact, the model likens mental files (i.e. singular term concepts) to internal anchors in DRT (Recanati 2016: 174), which thus represent mind-internal information about mind-external entities.

[1] Social cognition studies also show that our minds internalise social practices (cf. Fiske & Taylor 2013). Thus, these theories’ appeal to social conventions as mind-external, possibly normative principles governing language use seems divorced from the current scientific understanding of these matters.

References

Acquaviva, Paolo. 2008. *Lexical Plurals*. Oxford: Oxford University Press.

Acquaviva, Paolo. 2019. Two studies on the internal syntax of complex names. *Italian Journal of Linguistics* 31(2). 3–36. DOI: 10.26346/1120-2726-137

Acquaviva, Paolo. 2022. Word Meaning: a linguistic dimension of conceptualization. *Synthese* 200. 426–461. https://doi.org/10.1007/s11229-022-03910-9

Asher, Nicholas. 2011. *Lexical Meaning in Context: A Web of Words*. Oxford: Oxford University Press.

Buscaldi, David. 2011. *Toponym Disambiguation in Natural Language Processing*. University of Valencia Dissertation.

Busse, Dietrich. 2012. *Frame-Semantik: ein Kompendium*. Berlin: Walter de Gruyter.

Canter, David. 2012. *Facet theory: Approaches to social research*. Springer Science & Business Media.

Chen, Hao, Maria Vasardani & Stephan Winter. 2018. Georeferencing places from collective human descriptions using place graphs. *Journal of Spatial Information Science* 17(1). 31–62. http://dx.doi.org/10.5311/JOSIS.2018.17.417

Chierchia, Gennaro. 1998. Reference to Kinds across Languages. *Natural Language Semantics* 6(4). 339–405. DOI: https://doi.org/10.1023/A:1008324218506

Chierchia, Gennaro. 2010. Mass nouns, vagueness, and semantic variation. *Synthese* 174(1). 99–149. DOI: https://doi.org/10.1007/s11229-009-9686-6

Coates, Richard. 2017. The meaning of names: A defence of the pragmatic

theory of properhood (TPTP) addressed to Van Langendonck, Anderson, Colman and McClure. *Onoma* 52(1). 7–26. DOI:10.34158/ONOMA.52/2017/1

Cooper, Robin. 2017. Adapting Type Theory with Records for Natural Language Semantics. In Stergios Chatzikyriakidis & Zhaohui Luo (Eds.), *Modern Perspectives in Type-Theoretical Semantics*. Springer, Cham. https://doi.org/10.1007/978-3-319-50422-3_4

Cornilescu, Alexandra. 2007. On classifiers and proper names. *Bucharest Working Papers of Linguistics* 9(1). 61–75. https://doaj.org/article/fbbb0571f0af4e0caf42aec93ca1a6dc

Cruse, Alan D. 2000. *Meaning in language. An introduction to semantics and pragmatics*. Oxford: Oxford University Press.

Cumming, Stephanie. 2019. Names. In Edwin Zalta (Ed.), *The Stanford Encyclopedia of Philosophy* (Fall 2019 Edition).https://plato.stanford.edu/archives/fall2019/entries/names/

den Dikken, Marcel. 2006. *Relators and linkers: The syntax of predication, predicate inversion, and copulas*. Cambridge, MA. The MIT Press.

Derungs, Curdin & Ross Purves. 2014. From text to landscape: Locating, identifying and mapping the use of landscape features in a Swiss Alpine corpus. *International Journal of Geographical Information Science* 28(6). 1272–1293. doi:10.1080/13658816.2013.772184

Evans, Vyvian. 2015. A Unified Account of Polysemy within LCCM Theory. *Lingua* 152(6). 201–224. https://doi.org/10.1016/j.lingua.2014.12.002

Fellbaum, Christiane (Ed.). 1998. *WordNet: An electronic lexical database*. Cambridge, MA: MIT Press.

Fillmore, Charles J. 1982. Frame semantics. In Linguistic Society of Korea (ed.), *Linguistics in the morning calm*, 111–137. Seoul: Hanshin Publishing.

Fillmore, Charles J. & Collin Baker. 2010. A frames approach to semantic analysis. In Bernd Heine & Heiko Narrog (eds.), *The Oxford handbook of linguistic analysis,* 313–340. Oxford, UK: Oxford University Press. DOI: 10.1093/oxfordhb/9780199544004.013.0013

Fiske, Susan & Shelley Taylor. 2013. *Social Cognition: From brains to culture*. Riverside, CA: SAGE publications.

Fodor, Jerry & Zenon Pylyshyn. 2014. *Minds without Meanings: An essay on the Content of Concepts*. Cambridge, MA: the MIT Press.

Ganter, Bernhard & Rudolf Wille. 1998. *Formal Concept Analysis: Mathematical Foundations*. Berlin: Springer-Verlag.

Gieryn, Thomas F. 2000. A space for place in sociology. *Annual Review of Sociology* 26(1). 463–496. doi:10.1146/annurev.soc.26.1.463

Geurts, Bart, David I. Beaver & Emar Maier. 2020. Discourse Representation Theory. In Edward N. Zalta (Ed.), *The Stanford Encyclopedia of Philosophy.* https://plato.stanford.edu/archives/spr2020/entries/discourse-representation-theory/

Guizzardi, Giancarlo. 2005. *Ontological foundations for structural conceptual models*. Enschede: Telematics Instituut.

Hanks, Patrick. 2013. *Lexical analysis: Norms and exploitations*. Cambridge: the MIT Press.

Hanks, Patrick. 2000. Do word meanings exist? *Computers and the humanities.* 34(1). 205–215.

Haselbach, Boris. 2017. *Ps at the interfaces: On the Syntax, Semantics and Morphology of Spatial prepositions in German.* University of Stuttgart dissertation.

Haspelmath, Martin. 2019. Differential place marking and differential object marking. *Language Typology and*

Universals (STUF) 72(3). 313–334. doi:10.1515/stuf-2019-0013.

Heller, Daphna & Sarah Brown-Schmidt. 2023. The Multiple Perspectives Theory of Mental States in Communication. *Cognitive Science* 47(7). 1–32. https://doi.org/10.1111/cog.s13322

Hill, Linda. 2006. *Georeferencing: The geographic associations of information.* Cambridge, MA: the MIT Press.

Kamp, Hans. 2015. Using Proper Names as Intermediaries Between Labelled Entity Representations. *Erkenntnis* 80(2). 263–312.DOI:https://doi.org/10.1007/s10670-014-9701-2

Kamp, Hans. 2021. Sharing real and fictional reference. In Maier, Emar & Andreas Stokke, (Eds.) *The language of fiction*, 51–87. Oxford: Oxford University Press.

Kamp, Hans. 2022. The links of causal chains. *Theoria* 88(2). 296–325. Available from:https://doi.org/10.1111/theo.12381

Kamp, Hans, Joseph van Genabith & Uwe Reyle. 2011. Discourse Representation Theory. In Dov Gabbay & Franz Gunthner (eds.), *Handbook of Philosophical Logic 15*, 125–394. Dordrecht: Kluwer. DOI: 10.1007/978-94-007-0485-5_3

Kearns, Katie. 2006. Lexical semantics. In Baas Aarts & Andrea McMahon (Eds.), *Handbook of English Linguistics*, 557–580. Malden, MA/Oxford: Blackwell.

Kijania-Placek, Katarzyna. 2021. Names of Places. *Semiotica* 240(1). 187–210. https://doi.org/10.1515/sem-2021-0020

Köhnlein, Björn. 2015. The morphological structure of complex place names: The case of Dutch. *Journal of Comparative Germanic Linguistics* 18(1). 183–212. https://doi.org/10.1007/s10828-015-9075-0

Kracht, Marcus. 2002. On the semantics of locatives. *Linguistics and Philosophy* 25(1).57–132. https://doi.org/10.1023/A:1014646826099

Kracht, Marcus. 2004. Against the Feature Bundle Theory of Case. In Ellen Brandner and Heike Zinsmeister (Eds.), *New Perspectives on Case Theory*, 165–190. Stanford, CA: CSLI Publications.

Landman, Fred. 1991. *Structures for Semantics*. Dordrecht: Kluwer.

Levinson, Stephen C. & David Wilkins. (Eds.). 2006. *Grammars of Space: Explorations in Cognitive Diversity. Language, Culture, and Cognition 6*. Cambridge; New York: Cambridge University Press.

Löbner, Sebastian. 2011. Concept types and determination. *Journal of Semantics* 28(3).279–333. https://doi.org/10.1093/jos/ffq022

Löbner, Sebastian. 2014. Evidence for frames from natural language. Gamerschlag Thomas, Doris Gerland, Rainer Osswald & Wiebke Petersen (Eds.). 2014. *Frames and concept types. Applications in language and philosophy*, 23–68. Heidelberg: Springer. DOI: https://doi.org/10.1007/978-3-319-01541-5_2

Löbner, Sebastian. 2021. Frames at the Interface of Language and Cognition. *Annual Review of Linguistics* 7(1). 261–284 DOI 10.1146/annurev-linguistics-042920-030620

Mocnik, Franz-Benjamin. 2022. Putting Geographical Information Science in Place – Towards Theories of Platial Information and Platial Information Systems. *Progress in Human Geography* 46(3). 798–828.

Murphy, Marie L. 2010. *Lexical Meaning*. Cambridge University Press.

Pollard, Carl, and Ivan A. Sag. 1994. *Head-driven phrase structure grammar*. Chicago/London: The University of Chicago Press.

Riemer, Nicholas. 2010. *Introducing Semantics*. Cambridge: Cambridge University Press.

Pietroski, Paul. 2018. *Conjoining Meanings: Semantics without truth values*. Oxford: Oxford University Press.

Pustejovsky, James. 1995. *The generative lexicon*. Cambridge, MA: MIT Press.

Pylyshyn, Zenon. 2003. *Seeing and Visualizing: It's Not What You Think*. Cambridge, MA: MIT press.

Recanati, François. 1993. *Direct reference: From language to thought.* Oxford: Blackwell.

Recanati, François. 2012. *Mental Files*. Oxford: Oxford University Press.

Recanati, François. 2016. *Mental Files in Flux*. Oxford: Oxford University Press.

Recanati, François. 2021. Fictional reference as simulation. In Maier, Emar & Andreas Stokke, (Eds.) *The language of fiction*, 30–50. Oxford: Oxford University Press.

Rybka, Konrad. 2015. *The linguistic encoding of landscape in Lokono*. Ph.D. Dissertation, Raboud University Nijmegen.

Sag, Ivan A., Thomas Wasow & Emily M. Bender. 2003. *Syntactic theory: A formal introduction, 2nd ed.* Stanford, CA: CSLI Publications.

Sag, Ivan A., Hans C. Boas & Paul Kay. 2012. Sign–Based Construction Grammar: an informal synopsis. In Hans C. Boas & Ivan A. Sag (Eds.), *Sign–Based Construction Grammar*, 69–189. Stanford, CA: CSLI Publications.

Scannell, Leila, & Robert Gifford. 2010. The relations between natural and civic place attachment and pro-environmental behavior. *Journal of Environmental Psychology* 30 (5). 289–297.

Seamon, David. 2018. *Life takes Place: Phenomenology, lifeworlds and place-making*. London: Routledge.

Tenbrink, Thora. 2015. Cognitive discourse analysis: Accessing cognitive representations and processes through language data. *Language and Cognition* 7(1). 98–137.

Tenbrink, Thora. 2020. The language of place: towards an agenda for linguistic platial cognition research. In R. Westerholt & F.-B. Mocnik. (eds.), *Proceedings of the 2nd International Symposium on Platial Information Science* (PLATIAL'19), 5–12. DOI: 10.5281/zenodo.3628849.

Ursini, Francesco-Alessio. 2011. Space and the vision-language interface: A model-theoretic approach. *Biolinguistics* 5(3). 170-225.

Ursini, Francesco-Alessio & Giuseppe Samo. 2022. Names for urban places and conceptual taxonomies: The view from Italian. *Spatial Cognition & Computation* 22(3–4). 264–292. DOI: 10.1080/13875868.2021.1954186

Williams, Mai, Werner Kuhn & Marco Painho. 2012. The influence of landscape variation on landform categorization. *Journal of Spatial Information Science* 5(1). 51–73. doi:10.5311/JOSIS.2012.5.107

Zhou, Sha, Stephan Winter, Maria Vasardani & Shunping Zhou. 2016. Place descriptions by landmarks. *Journal of Spatial Science* 62(1). 47–67. https://doi.org/10.1080/14498596.2016.1196623
